# Income, consumer preferences, and the future of livestock-derived food demand

**DOI:** 10.1016/j.gloenvcha.2021.102343

**Published:** 2021-08-23

**Authors:** Adam M. Komarek, Shahnila Dunston, Dolapo Enahoro, H. Charles J. Godfray, Mario Herrero, Daniel Mason-D’Croz, Karl M. Rich, Peter Scarborough, Marco Springmann, Timothy B. Sulser, Keith Wiebe, Dirk Willenbockel

**Affiliations:** a International Food Policy Research Institute, Washington DC, USA; b The University of Queensland, School of Agriculture and Food Sciences, Gatton, Qld 4343 Australia; c Policies, Institutions and Livelihoods Program, International Livestock Research Institute (ILRI), P.O. Box 30709, Nairobi 00100, Kenya; d Oxford Martin Programme on the Future of Food, Oxford Martin School, University of Oxford, 34 Broad Street, Oxford OX1 3BD, UK; e Department of Zoology, University of Oxford, South Parks Road, Oxford OX1 3PS, UK; f Commonwealth Scientific and Industrial Research Organisation, Australia; g Nuffield Department of Population Health, University of Oxford, Old Road Campus, Headington, Oxford OX3 7LF, UK; h Institute of Development Studies, University of Sussex, Brighton, UK; i Oklahoma State University, Ferguson College of Agriculture, Department of Agricultural Economics, Stillwater, OK 74078, USA; j Department of Global Development, College of Agriculture and Life Sciences and Cornell Atkinson Center for Sustainability, Cornell University, Ithaca, NY 14853, USA

**Keywords:** Animals, Commodity prices, Demand, Income elasticity, Livestock

## Abstract

In recent decades there has been a sustained and substantial shift in human diets across the globe towards including more livestock-derived foods. Continuing debates scrutinize how these dietary shifts affect human health, the natural environment, and livelihoods. However, amidst these debates there remain unanswered questions about how demand for livestock-derived foods may evolve over the upcoming decades for a range of scenarios for key drivers of change including human population, income, and consumer preferences. Future trends in human population and income in our scenarios were sourced from three of the shared socioeconomic pathways. We used scenario-based modeling to show that average protein demand for red meat (beef, sheep, goats, and pork), poultry, dairy milk, and eggs across the globe would increase by 14% per person and 38% in total between the year 2020 and the year 2050 if trends in income and population continue along a mid-range trajectory. The fastest per person rates of increase were 49% in South Asia and 55% in sub-Saharan Africa. We show that per person demand for red meat in high-income countries would decline by 2.8% if income elasticities of demand (a partial proxy for consumer preferences, based on the responsiveness of demand to income changes) in high-income countries decline by 100% by 2050 under a mid-range trajectory for per person income growth, compared to their current trajectory. Prices are an important driver of demand, and our results demonstrate that the result of a decline in red meat demand in high-income countries is strongly related to rising red meat prices, as projected by our scenario-based modeling. If the decline in the income elasticity of demand occurred in all countries rather than only in high-income countries, then per person red meat demand in high-income countries would actually increase in 2050 by 8.9% because the income elasticity-driven decline in global demand reduces prices, and the effect of lower prices outweighs the effect of a decline in the income elasticity of demand. Our results demonstrate the importance of interactions between income, prices, and the income elasticity of demand in projecting future demand for livestock-derived foods. We complement the existing literature on food systems and global change by providing quantitative evidence about the possible space for the future demand of livestock-derived foods, which has important implications for human health and the natural environment.

## 1 Introduction

Since the 1980s there has been a continued increase in the global demand for livestock-derived foods (Clonan et al., 2016), collectively beef, sheep, goats, pork, poultry, dairy milk, and eggs in our study. Although livestock-derived food demand continues to increase in most countries as part of the global nutrition transition (Popkin and Gordon-Larsen, 2004), demand in some countries has started to plateau or even decline, especially in developed countries (Godfray et al., 2018; Bentham et al., 2020). The livestock sector has both positive and negative effects on human and natural systems (Thornton, 2010; Mehrabi et al., 2020). Plant-based diets generally have less negative impacts on the natural environment (hereinafter environment) compared to animal-based diets (de Vries and de Boer, 2010; Sabaté and Soret, 2014; Clark and Tilman, 2017). Globally the nutrition transition has seen diets shift away from being high in whole grains to containing more saturated fats, added sugar, refined carbohydrates, and livestock-derived foods (Popkin, 2006). This shift has been linked to a higher prevalence of non-communicable diseases, for example the consumption of processed meat and red meat has been associated with an increased risk of cardiovascular diseases (Sinha et al., 2009; Chen et al., 2013; Yang et al., 2016), type 2 diabetes (Feskens et al., 2013), and colorectal cancer (Bouvard et al., 2015). Livestock-derived foods can make important contributions to human diets, especially for pastoralists (Galvin, 1992; Sellen, 1996). Livestock-derived food consumption can have positive contributions to human health (hereinafter health), such as reduced child stunting in low- and middle-income countries (Headey et al., 2018) although evidence is still being debated (Shapiro et al., 2019), and providing micronutrients that are difficult to obtain in adequate quantities from plant-based diets alone, such as vitamin B12 (Murphy and Allen, 2003). The livestock sector also provides a source of income and employment for hundreds of millions of people (Herrero et al., 2009; Mehrabi et al., 2020). Given this widespread and multidimensional effect of livestock-derived food demand on human and natural systems, it is important to understand how factors of demand, such as income, price, and consumer preferences, interact with each other and contribute to livestock-derived food demand.

Our current study aims to shed light on unanswered questions about how livestock-derived food demand may evolve over the upcoming decades, which has relevance to ongoing debates on how livestock production and consumption affect health and the environment. The objectives of our current study were to 1) project per person and total demand for livestock-derived foods across the globe, disaggregated by regions and countries, out to the year 2050 for a range of population and income trajectories and 2) project how changes in the income elasticity of demand affect per person red meat demand in 2050 relative to 2020 levels. In our study, the word region refers to a group of countries, not a sub-national unit within one country. The income elasticity of demand describes the responsiveness of demand to income changes. We used a multimarket global economic model to simulate demand under a range of what-if scenarios. Our intent was to explore demand projections under a range of scenarios, we did not attempt to predict demand. The essence of the multimarket model approach is to consider one sector, the agriculture sector in our case, and examine the factors influencing the supply and demand of multiple commodities within the one sector.

Several approaches have been used to project livestock-derived food demand at the scale of an individual consumer, country, region, or the globe. For individual consumers, studies have mostly used surveys to examine willingness-to-reduce meat consumption, rather than studying observed demand (Bryant and Barnett, 2018). Economic models have examined how factors such as population, income, and elasticities affect livestock-derived food demand (Valin et al., 2014; Popp et al., 2017; Bai et al., 2018; Enahoro et al., 2018) and long-term trends in food demand and prices (Hertel et al., 2016). Others have combined accounting identities and exogenous assumptions on population and income growth with expert judgments (Alexandratos and Bruinsma, 2012; Desiere et al., 2018; FAO, 2018), extrapolated historical income trends (Tilman et al., 2011; Bodirsky et al., 2015; Davis et al., 2016), and predicted demand changes by dynamically adjusting income elasticities of demand (Bijl et al., 2017). At the global scale, Eker et al. (2019) examined the behavioral change needed to achieve dietary shifts through the use of a psychological framework in a diet change model. In their study, people change diet choices based on climate and health risks, and social norms. Although approaches vary, most of the above studies conclude that livestock-derived food demand will increase at rates that vary by commodity and country. Several studies have compared *ex-ante* model projections with actual demand (once realized). For example, demand projections for livestock-derived foods in 2020 (Delgado et al., 2001) were closer to actual demand for pork than poultry meat, and were, in general, quite prescient for other livestock-derived foods (Herrero et al., 2018). And although developing countries have contributed the most to global growth in livestock-derived food demand, this growth has been concentrated in a few countries, such as Brazil, China, India, and Indonesia (Pica-Ciamarra and Otte, 2011).

Only a few global-scale studies have examined income elasticities for livestock-derived foods within an economic framework, such as Valin et al. (2014), despite income elasticities (and consumer preferences more generally) being critical in affecting food demand (Unnevehr et al., 2010; Gao, 2012; Hertel et al., 2016; Jones et al., 2016). Our study complements analyses such as Valin et al. (2014) by explicitly examining a range of changes in the income elasticity of demand for red meat. It also complements Eker et al. (2019) as we examine how demand would be affected by changes in income and subsequent feedback effects on commodity prices. Several studies have examined the health and environmental implications of alternative diets including reduced demand for livestock-derived foods (Springmann et al., 2016; Popp et al., 2017; Weindl et al., 2017; Willett et al., 2019), and our study complements these studies by providing an analysis of what livestock-derived food demand would be under a range of trajectories for income, population and the income elasticity of demand for individual livestock-derived foods and specific regions, taking into account endogenous commodity prices. We addressed uncertainty by using scenarios to explore the future of livestock-derived food demand under a range of trajectories for income, population, and the income elasticity of demand. SI Section 1 describes the role of uncertainty in our study.

## 2 Methods

### 2.1 Model

We simulated scenarios for how changes in human population size, per person gross domestic product (hereinafter income), and the income elasticity of demand would affect livestock-derived food demand at the country scale. The simulation unit for demand in our study is a commodity in a country in a year. The simulations were conducted in version 3 of the International Model for Policy Analysis of Agricultural Commodities and Trade (IMPACT) (Robinson et al., 2015). The model is a process-based deterministic model. The core of the model is a global partial equilibrium multimarket economic model that simulates national and international agricultural commodity markets. The model is solved to ensure that for a commodity global supply equals global demand at the end of every year. The model simulates demand, supply, international trade (exports and imports), and prices for agricultural commodities using a series of structural equations. Commodity prices are endogenous in the model. The commodities we examined included six livestock-derived foods: beef, sheep (& goat meat), pork, poultry (meat), eggs, and dairy milk. In our study, sheep includes sheep meat and goat meat, and hereinafter we refer to meat from sheep and goats as sheep. Quantities of meat are for dressed meat or carcass weight. Demand in our study is for agricultural commodities (such as beef); rather than for the demand for specific products derived from the commodity (such as offals or cuts from beef).

Total household demand for each livestock-derived food in a country is a function of total population, per person income, the price of the livestock-derived food commodity, prices of competing commodities, the income elasticity of demand and the price elasticity of demand. SI Section 1 provides technical details on how the model simulates livestock-derived food demand and supply, including the formation of endogenous commodity prices. Income and price are important factors of demand; however, other factors also play an important role in explaining demand (Unnevehr et al., 2010). As an example, Tonsor and Marsh (2007) estimated that about 75% of the variability in meat demand in the USA is driven by factors other than income and meat prices. Some of these other factors include consumer attitudes towards the health and the environmental implications of livestock-derived food production and consumption, attitudes towards food safety, the visual appearance of food (such as red meat marbling), convenience of food preparation, and animal welfare concerns, among others. These other factors are often called consumer preferences (hereinafter preferences) (Unnevehr et al., 2010).

Consumer preferences tend to change over time, for example for meat in the USA (Moschini, 1991), and these preferences are connected to elasticities. We assumed in our model that changes in the income elasticity of demand (an exogenous parameter in our model) are a partial proxy for changes in preferences. SI Section 1 provides details on the underlying theory of consumer behavior relevant to the connection between consumer preferences and the income elasticity of demand. The income elasticity of demand is the percentage change in quantity of a commodity demanded if income changes by 1%, all other factors equal. The price elasticity of demand is the percentage change in commodity demand if the price of that commodity changes by 1%, again all other factors equal. Demand in the model is determined by the model structure and data, and demand is consistent with Engel’s Law (Zimmerman, 1932) and Bennett’s Law (Bennett, 1941).

Data for population, income, and elasticities came from several sources. Data for total population and total income by country out to 2050 came from the shared socioeconomic pathways (SSP) Database - Version 2.0 (Riahi et al., 2017), as detailed in Section 2.2. The price and income elasticities of demand were initially based on a USDA (1998) dataset, with periodic revisions informed by observed demand trends (Delgado et al., 2001; Seale et al., 2003; Regmi and Seale, 2010; Green et al., 2013; Mead et al., 2014; Cornelsen et al., 2015). The updating of data used in the model is an ongoing process and is also informed by the results of expert-judgment scenarios (Alexandratos and Bruinsma, 2012; Bodirsky et al., 2015) and participation in integrated assessments and multi-model analyses (Rosegrant et al., 2009; Valin et al., 2014; von Lampe et al., 2014; Stehfest et al., 2019).

**Fig. 1:**
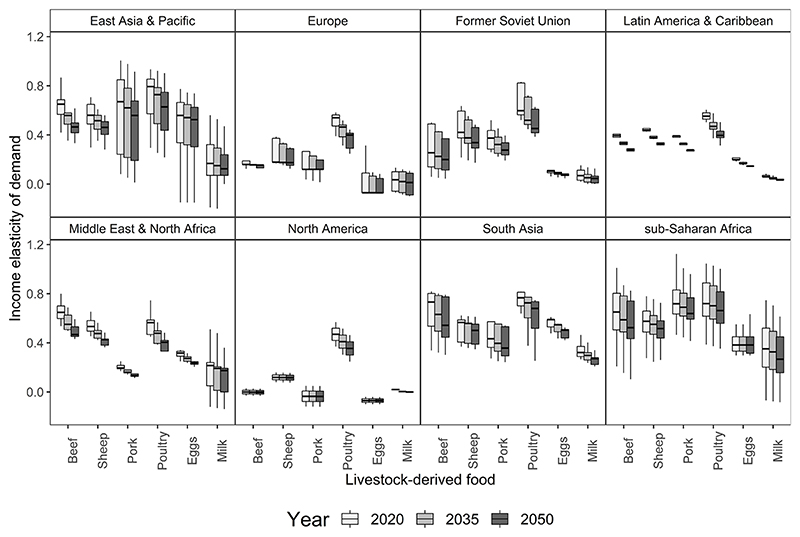
Country-specific income elasticities of demand for livestock-derived foods by region used in the model. Elasticity is the % change in quantity demanded if income changes by 1%. Boxes indicate the interquartile range (IQR). The upper whisker extends from the third quartile upper hinge of the box to the largest value no further than 1.5 × IQR from the upper hinge. The lower whisker extends from the first quartile lower hinge of the box to the smallest value at most 1.5 × IQR from the lower hinge. Outliers are not plotted for clarity. The line dividing each box shows the median.

Fig. 1 reports the country-scale income elasticity of demand used in our model for each livestock-derived food by region. We grouped all countries in the world into eight regions: 1) East Asia & Pacific; 2) Europe; 3) Former Soviet Union; 4) Latin America & Caribbean; 5) Middle East & North Africa; 6) North America; 7) South Asia; and 8) sub-Saharan Africa (Robinson et al., 2015). Data in Fig. 1 are the reference case income elasticities used directly in our simulations (Section 2.2), and these data are exogenous parameters. We used a single income elasticity in each year for each commodity-country combination.

The general long-term downward trend for income elasticities in our model reflects the widespread empirical finding that the income elasticity of demand generally falls as income increases (Hudson and Vertin, 1985; Cirera and Masset, 2010; Bijl et al., 2017; Muhammad et al., 2017; Colen et al., 2018; Desiere et al., 2018; Zhou et al., 2020). There is considerable variation in income elasticities across specific commodities, income groups, regions, and on a year-on-year basis (Fig. SI.1 and Fig. SI.2). The general downward trend of income elasticities used in our model (as income increases over time) is also consistent with Engel’s Law, which has widespread empirical support (Houthakker, 1957; Unnevehr et al., 2010; Clements and Si, 2018). In general, our elasticities for sheep and pork are lower than the elasticities for beef—similar to existing estimates (Gallet, 2010), while poultry elasticities tend to be higher than red meat elasticities (Fig. 1), as reported in existing studies (Hudson and Vertin, 1985). Our elasticities are generally lower in higher-income countries than in lower-income countries, similar to existing studies (Cranfield et al., 2002; Muhammad et al., 2017). The price elasticity of demand for each livestock-derived food is negative in our model (Fig. SI.3).

Although our study explores the future demand for livestock-derived foods, our multimarket model equates global supply with global demand for each commodity. Therefore, supply is an important part of the model, especially for determining commodity prices. Supply is a product of animal numbers and yield per animal. The total number of animals is a function of the previous year’s animal numbers and its exogenous growth trend, animal prices, and prices for feed grain crops. Yield per animal is based on a non-price (exogenous) productivity growth rate. SI Section 1 provides additional details on the demand and supply equations and accompanying input data parameters.

### 2.2 Simulation scenarios

#### 2.2.1 Overview of scenarios

We simulated livestock-derived food demand at the country-scale between the year 2020 and the year 2050 under a range of what-if scenarios (Table 1). We first simulated three SSP reference case scenarios that used trajectories for income and population growth from three of the SSP narratives: (1) the mid-range SSP2, (2) SSP1 that is relatively optimistic compared with SSP2, and (3) SSP3 that is relatively pessimistic compared with SSP2. In all three of these reference case scenarios, we used the reference case elasticities that assume no change to our current assumptions about the temporal trajectory for the income elasticity of demand for livestock-derived foods (Fig. 1). We then examined the consequences of a decline in the income elasticity of demand for red meat for each of the three SSP income and population trajectories (Table 1) looking at both the case of a global reduction and one confined to high-income countries based on the World Bank’s Country Group classification (World Bank, 2020). The decline in income elasticities were implemented through a change from the trajectory of reference case elasticities. In total we ran 15 what-if scenarios: five trajectories for income elasticities (reference case and four red meat income elasticities) and all five trajectories for income elasticities were simulated for each of the three SSP income and population combinations (SSP1, SSP2, and SSP3).

##### 2.2.1.1 SSP reference case scenarios

We used the population and income data associated with three SSP narratives in our reference case scenarios. We did not include the other factors in each SSP narrative in our reference case scenarios, such as globalization or land-use regulation. This allowed us to focus on the interaction of changes in income and the income elasticity of demand. Any reference to an SSP in our study is only for the income and population in that SSP narrative. In SSP1 income growth is higher and population growth is lower compared to SSP2. In SSP3 income growth is lower and population growth is higher compared to SSP2 (Table 1). The SSPs are reference pathways that assume no climate change or climate impacts, and no new climate policies (O’Neill et al., 2014). Data on population and its change in each SSP were compiled and developed by the International Institute for Applied Systems Analysis (IIASA) (Kc and Lutz, 2017) and we used the population trajectories of IIASA in all our scenarios. Data on income and its change over time were based on the Organisation for Economic Cooperation and Development (OECD) interpretations of the SSPs (Dellink et al., 2017). Projected changes in agricultural production in IMPACT over time affected the income levels used in all 15 simulations (SI Section 1: GLOBE to IMPACT link), therefore income levels in our model slightly deviate from the income trajectories specified by the OECD.

##### 2.2.1.2 Alternative red meat income elasticities scenarios

We simulated 12 what-if scenarios for declines in the income elasticity of demand for red meat. The simulations considered what would happen if the income elasticities of red meat demand declined at rates faster than those in the reference case scenario (Fig. 1) under each SSP income and population combination. After running reference case scenarios for SSP1, SSP2, and SSP3 income and population with reference case elasticities, we simulated four alternative scenarios for each SSP income and population. For our red meat income elasticities scenarios, we followed the one-(factor)-at-a-time approach (Pannell, 1997; Pianosi et al., 2016). In this approach in all red meat income elasticities scenarios we only changed the livestock-derived food income elasticities and the income elasticities for all other commodities remained the same as in the reference case scenario for each SSP. In the red meat income elasticities scenarios, the primary effect (direct first-round effect) of a decline in the income elasticity of demand would be a decline in expenditures on red meat, which would be spent on other, non-agricultural, product categories. A secondary effect would be how changes in the demand for livestock-derived foods affect the prices of all commodities. As an example, if the income elasticity of beef demand was 0.4, a 1% increase in income would lead to a 0.4% increase in beef demand, and in our scenarios if the elasticity declined by 50% to 0.2, a 1% increase in income would lead to a 0.2% increase in beef demand. As a further example, if the parameter for the income elasticity of demand in 2020 was 0.6 and our reference case scenario assumed in 2050 the income elasticity of demand was 0.3, then in a scenario for a 100% decline in the income elasticity of demand the income elasticity of demand in 2050 would be zero. With an income elasticity of zero, a change in income has no effect on the quantity demanded (assuming all other factors are constant). In our what-if scenarios, we quantify what would happen if the income elasticity of demand changes but do not answer the question of why the income elasticity of demand might change.

The motivation for focusing on declines in the income elasticity of demand for red meat was that red meat has received attention for its negative effects on health and the environment (Introduction Section 1), relative to some other foods. In two of these red meat income elasticities scenarios, we explored a decline in the income elasticity of demand for red meat in high-income countries only, and in the other two red meat income elasticities scenarios, we explored a decline in the income elasticity of demand for red meat in all countries of the world. Using data from the SSP database for 2020, high-income countries had 16% of the world’s total population and 46% of the world’s total income. We focused on high-income countries because of the high levels of consumption in these countries (Clonan et al., 2016) and because high-income countries are generally closer to the final stage of the nutrition transition (Popkin and Gordon-Larsen, 2004). In this final stage behavioral change occurs that may reduce red meat consumption as consumers may have a greater concern for or become more aware of issues such as the effect of red meat consumption on their health or the environment, along with animal ethics and welfare concerns.

##### 2.2.1.3 Protein calculations

We reported the quantities of simulated demand for each individual livestock-derived food in total quantities (metric tons) and per person quantities (kg). We converted these quantities of food into a quantity of protein using the protein content of each livestock-derived food. The protein content (grams protein per 100 g food) and calorie content (calories per 100 g food) of each commodity was derived from the Global Expanded Nutrient Supply (GENuS) Model (Smith et al., 2016) for consistent values in 2011 (Table SI.1). Although our primary interest was livestock-derived food demand in kilograms of food, we converted kilograms of food to protein aggregated over the six livestock-derived foods and also over the three red meats to provide an indicator in a consistent unit. Each kilogram of livestock-derived food has a different nutrient content. We then used these protein contents to compute the total demand for protein from livestock-derived foods.

## 3 Results

### 3.1 Reference case scenarios for income and population

**Fig. 2:**
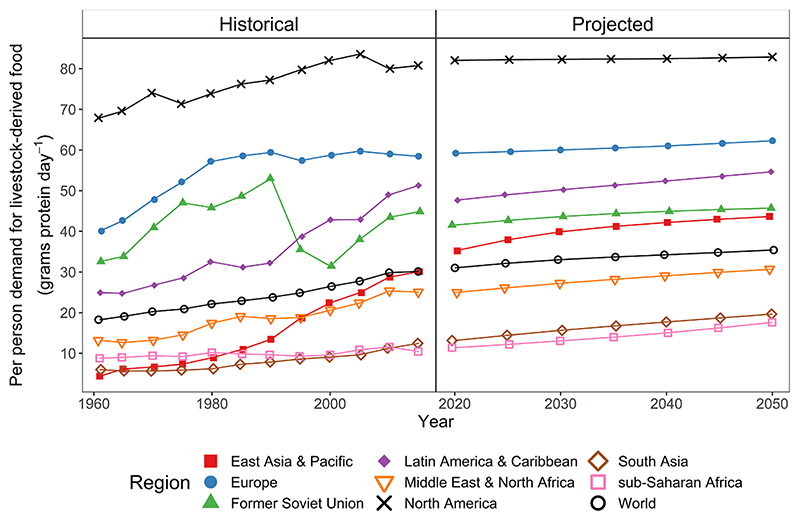
Historical and projected trends in per person demand for protein aggregated over six livestock-derived foods (beef, sheep & goat meat, pork, poultry meat, dairy milk, and eggs) by region and year. Historical data from Food Balance Sheets (FAO, 2020). Projected data simulated using income and population from shared socioeconomic pathway 2 and the reference case elasticities.

The global average demand for livestock-derived food per person has increased from 22 g protein day^−1^ in 1980 to 30 g protein day^−1^ in 2010, with variation among regions in both absolute per person demand and the rate of change in demand (Fig. 2). For example, per person demand was consistently highest in North America and Europe and was consistently lowest in South Asia and sub-Saharan Africa. The East Asia & Pacific region has the fastest percent growth in per person demand with a 222% increase between 1980 and 2010.

Using SSP2 income and population data (the mid-range SSP) for the reference scenario for future projections, projections of per person demand in 2050 suggest that the fastest growth will be in South Asia (13 g protein day^−1^ in 2020 to 20 g protein day^−1^ in 2050) and sub-Saharan Africa (11 g protein day^−1^ in 2020 to 18 g protein day^−1^ in 2050), albeit from lower initial levels relative to other regions. These aggregate demand values masked the differences in demand for individual livestock-derived foods and their trajectories at the region (Table 2) and country scale (Fig. SI.5). Although, the contribution of individual livestock-derived foods to total protein demand has remained stable over time (Fig. SI.6). To further highlight trends in demand, Fig. SI.7 highlights the wide range of trajectories for per person demand for each livestock-derived food by region between 1961 and 2013 for historical data and between 2005 and 2050 for projected data. For example, fast projected growth in livestock-derived food demand in sub-Saharan Africa of 55% between 2020 and 2050 (Fig. 2) was related to sub-Saharan Africa having the fastest growth in beef and pork demand globally. At the country scale (Fig. SI.5), we project a continuation of rapid annual growth in poultry demand in India of 4.33% would occur under SSP2. We also project that there would be declines in demand for some livestock-derived foods between 2020 and 2050 under SSP2, especially for dairy milk and eggs in some high-income countries (Fig. SI.5). Across all livestock-derived foods and countries, the annual growth rate in per person demand between 2020 and 2050 (Fig. SI.5) had a positive skew (Table SI.2) with 75% of countries having an annual growth rate of less than 2.03%. Per person pork demand between 2020 and 2050 rises in seven of the eight regions but falls at the global scale, and this perhaps counterintuitive-arithmetic phenomenon is commonly called Simpson’s paradox (SI Section 2).

**Fig. 3:**
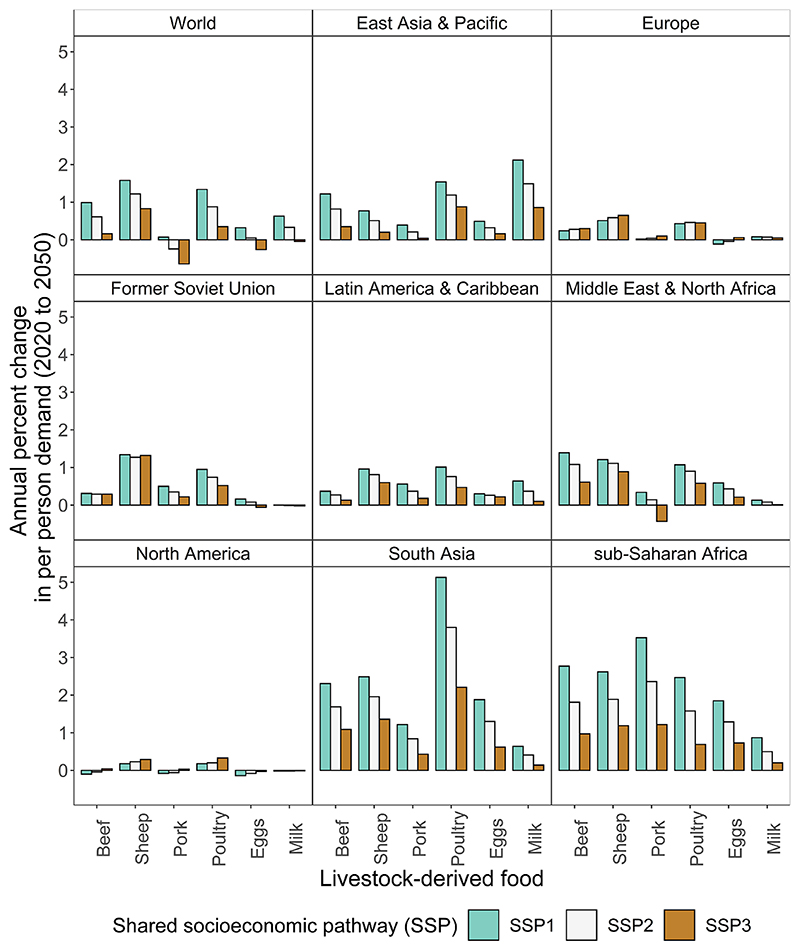
Annual percent changes in per person demand for livestock-derived food by region, simulated using income and population from three shared socioeconomic pathways and the reference case elasticities. Sheep includes sheep and goat. Percent change based on compound annual growth rate.

At the global level, faster growth in income led to livestock-derived food demand increasing over time (SSP1 relative to SSP2), and slower growth in income led to per person demand decreasing over time (SSP3 relative to SSP2) (Fig. 3). At the region scale (in SSP3, relative to SSP2), slower growth in income across the globe led to an increase in per person demand in higher-income regions such as North America and Europe. This increase was because lower prices for livestock-derived foods prevailed because of reduced demand in lower-income countries, and demand growth was fastest in South Asia and sub-Saharan Africa (Fig. 3).

**Fig. 4:**
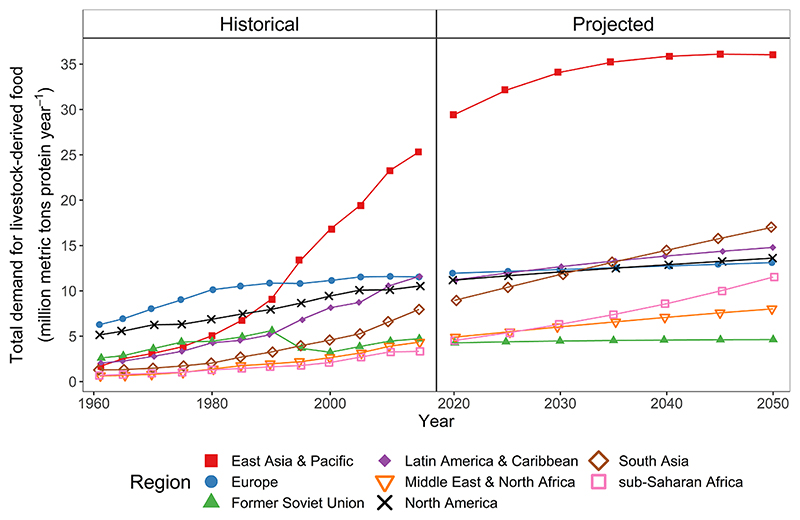
Historical and projected trend in total demand for protein aggregated over six livestock-derived foods (beef, sheep & goat meat, pork, poultry meat, dairy milk, and eggs) by region and year. Historical data from Food Balance Sheets (FAO, 2020). Projected data simulated using income and population from shared socioeconomic pathway 2 and the reference case elasticities.

Our projections suggested that total demand for livestock-derived foods would increase by 38% between 2020 and 2050 under SSP2. The total population size of each region has a major role in driving total demand, and East Asia & Pacific and South Asia had the largest projected total demand in 2050 (Fig. 4). Like per person demand, rates of change in total demand varied across regions and livestock-derived foods (Table SI.3 and Fig. SI.8). Under SSP2, between 2020 and 2050 total demand would rise by 155% in sub-Saharan Africa, from low levels in 2020, and by 8% in the Former Soviet Union, with increases in other regions between these two values. Historical data suggest between 1980 and 2010 East Asia & Pacific had the fastest total increase in livestock-derived food demand (360%), but the rate of increase between 2020 and 2050 in sub-Saharan Africa of 155% and in South Asia 91% is projected to be faster than in other regions. The share of total global red meat protein demand coming from high-income countries was 31% in 2020 and 26% in 2050.

### 3.2 Red meat income elasticities scenarios

Under SSP1 and SSP2 income and population trends with the reference case income elasticities, we project that average per person demand for red meat (sum of beef, sheep, and pork) will increase by 2050, relative to 2020, in high-, and low- and middle-income countries (Table 3). For SSP2, if income elasticities of demand for red meat declined such that their trajectory over time meant that they were 50% less in 2050 than in the reference case in 2050 in high-income countries, per person red meat demand in high-income countries would increase by 0.5% in 2050, relative to demand in 2020 of 25 g day^−1^ (beef 24.8 kg y^−1^, sheep 2.1 kg y^−1^, and pork 31.0 kg y^−1^) with a 100% decline leading to a 2.8% decline in per person red meat demand. The effect on red meat demand in low- and middle-income countries would be limited. However, if income elasticities for red meat were to decline in all countries in SSP2, then demand in high-income countries would increase by 2050, relative to demand in 2020. Income elasticities of demand are typically higher in low-and middle-income countries than in high-income countries (Fig. SI.2), and we discuss this perhaps counterintuitive result in the Discussion. Looking at the results for SSP1 and SSP3 in Table 3, as the rate of per person income growth increased (from the slower growth in SSP3 to the mid-range growth in SSP2 then to faster growth in SSP1) the change in per person demand between 2020 and 2050 increases for global demand and for demand in low-and middle-income countries in all red meat income elasticities scenarios. However, if the income elasticity of demand declines only in high-income countries then demand in high-income countries (% change 2020 to 2050) declines by 2.8% in SSP2, declines by 4.3% in the faster income growth SSP1, and declines by 1.5% in the slower income growth SSP3. These declines in red meat protein demand in high-income countries are because pork demand declines, despite beef and sheep demand increasing (Table SI.4). For the red meat income elasticities scenarios, a 50% or 100% decline in the income elasticity still means total demand increases in 2050 relative to 2020 (Table SI.4). There was a wide distribution of demand changes at the country-scale (Table SI.5) that underpin the averages in Table 3.

**Fig. 5:**
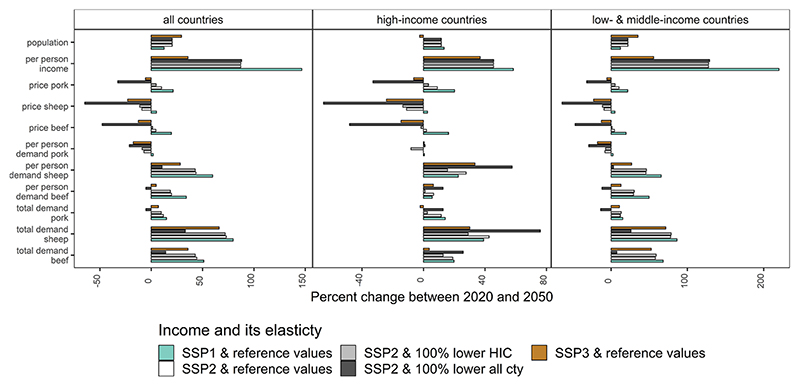
Red meat changes in demand (total and per person) and demand factors (income, price, and population). In the simulation model each year, the units for population are people, units for income are dollars per person, units for prices are dollars per kilogram of the commodity (pork, sheep, or beef), units for per person demand are kilograms per person, units for total demand are kilograms per country. Elasticities are the income elasticities of demand for red meat. Projected data simulated using income and population from three shared socioeconomic pathways (SSP). Countries grouped based on the World Bank’s 2020 Country Group classification. cty = countries and HIC = high-income countries. (For interpretation of the references to colour in this figure legend, the reader is referred to the web version of this article.)

Fig. 5 reports how income, population, and price affect red meat demand by country group for combinations of SSPs and income elasticities for red meat. If the income elasticity of demand for red meat declined in all countries (under SSP2), all other things equal, there will be a decline in demand, which will lead to a fall in beef prices. This price change has two effects, one to signal to producers to reduce their production, and two making red meat relatively cheaper, which can lead to a rebound effect in demand. This rebound effect contributes to an increase in beef demand in high-income countries (price effects are dominating the income effects). But this global decline in the income elasticity of demand would lead to a decrease in beef demand in low- and middle-income countries, where the income effects are expected to continue dominating the price effects. Between 2020 and 2050 for the reference case income elasticities, with income growth from SSP2 prices rise for beef and pork and prices fall for sheep, with income growth from SSP3 prices for all three red meats fall under SSP3, and with income growth from SSP1 prices for all three red meats rise (Fig. 5 for percentage change in price and Fig. SI.9 for price levels).

## 4 Discussion

We begin by comparing demand projections with projections from the existing literature, and then explain why livestock-derived food demand changes under different scenarios, focusing on the role of prices, income, and income elasticities. Valin et al. (2014) reported that 11 global economic models projected increases in global demand for livestock calories under SSP2 between 2005 and 2050 that ranged from 12 to 140% for per person demand and from 61 to 242% for total demand. IMPACT projections reported in Valin et al. (2014) estimated the global increase in demand for livestock calories between 2005 and 2050 at 25% for per person demand and 78% for total demand. We find that under the reference case scenario with SSP2, global demand for livestock-derived foods (in calories) is projected to increase between 2005 and 2050 by 20% for per person demand and by 70% for total demand. SI Section 1 briefly compares some of the data and assumptions in the 11 global economic models that help explain differences between model results. In addition to providing updated projections for protein demand, our results highlight how the effect of changes in income on demand is mediated by price effects.

Many factors influence per person livestock-derived food demand, some of which include per person income, income elasticities, and prices of livestock-derived foods, with total population changes contributing to total demand changes. Attributing the contribution of each factor to a change in demand is challenging as factors do not change in isolation and are inter-connected. Here we discuss two processes illustrated by our results, 1) how income elasticities affect livestock-derived food demand, and 2) how commodity prices mediate the effect of income on demand.

First, we examined how changes in the income elasticities for red meat affect its demand under a range of income levels. The differentiated response to a decline in the income elasticity of demand under each SSP is because with faster income growth under SSP1 prices rise faster (Fig. SI.9) than in SSP2 or SSP3, so demand declines by more in high-income countries as income growth rises, especially as income elasticities declined by 50% or 100% in the scenario, thereby muting the income response. If the income elasticity of demand for red meat declines in high-income countries only then demand in high-income countries would fall under conditions of faster income growth. But if the income elasticity of demand for red meat declines in all countries then the reduced global demand leads to a global decline in prices and this would increase demand in high-income countries, despite income elasticities declining. In high-income countries, with a decline in the income elasticity of demand for red meat of 100%, there is a decline in per person red meat demand over time because there is a reduced positive exogenous response to increased income over time that is less than the negative endogenous price effect from rising prices over time. Demand fell over time because the price effect outweighed the income effect.

The decline in per person demand for red meat following a 100% decline in income elasticities in high-income countries has relevance for human health; however, the 100% decline in income elasticities still means that total red meat consumption in high-income countries (and globally) increases between 2020 and 2050. And, if the income elasticity of demand for red meat declined by 100% in all countries per person demand for red meat would decrease by 18% in low- and middle-income countries and increase by 9% in high-income countries.

Second, our results from comparing demand under the three SSPs suggest that if income growth slows globally, which is the case in SSP3 (relative to SSP2), then demand in lower-income countries is expected to fall in 2050 under SSP3 compared to demand in 2050 under SSP2. This is because the direct income effect of a decline in income growth slowing livestock-derived food demand growth outweighs the positive effect lower prices have on demand. The income effect outweighs the price effect to see demand decline in lower-income countries, and income growth rates and income elasticity levels in these lower-income countries are generally greater than in higher-income countries. However, in higher-income countries as income growth slows globally under SSP3 (relative to SSP2) the slowdown in demand in lower-income countries results in a decline in global prices, which results in demand actually rising in higher-income countries in 2050 under SSP3 compared to demand in 2050 under SSP2. This rising demand is also related to lower income elasticities of demand in high-income countries compared with low- and middle-income countries, and demand is more sensitive to changes in income in low- and middle-income countries.

Our model-based result of falling livestock-derived food prices leading to increased livestock-derived food demand has been estimated in global *meta*-regressions (Green et al., 2013) and in beef retail markets in the USA (Capps, 1989). Our results suggest that per person income trajectories influence red meat prices, with slower per person income growth (SSP3) leading to price declines and faster per person income growth (SSP1) leading to price increases. The results for the mid-range per person income growth (SSP2) saw higher prices for beef and pork but lower prices for sheep. In the comparison of global economic models (Valin et al., 2014; von Lampe et al., 2014) four of the ten models (including three partial equilibrium models) saw increasing prices for ruminant meat by 2050, while the remaining six models saw livestock prices falling or remaining steady. Existing studies have also reported simulated agricultural prices out to the year 2050 using six global economic models for SSP1, SSP2, and SSP3 (Stehfest et al., 2019), and although prices do not rise in all model-SSP combinations, the major trend was for higher prices in 2050 than in 2010 or 2020. From historical data, the FAO Food Price Index shows a price spike in 1972–1974, then a decline and flattening in the 1990s, and an upturn with increased volatility since about 2000, including for meat and dairy, as reported in Brooks and Place (2019).

Our study has its limitations two of which include the aggregation of specific cuts and products into a generic commodity and data sources of elasticities and their estimation. At a more granular level of analysis, one might expect a gradual decline in demand for commodity (low value) cuts and offals. In developing countries, one might expect such a shift due to rising incomes that can allow consumption of higher-quality cuts, although in some contexts, this will be mediated by cultural preferences for specific products. In developed countries, a shift towards meat substitutes and lab-grown meats could lead to a substitution of such products for lower-quality meat, whereas demand for premium cuts could rise. However, the net effect of such changes is unclear given that various cuts are co-produced within the carcass. For elasticity data, we advocate for an increased focus on the improved estimation of elasticities including consistency with data sources (time series versus cross sectional), a topic that has been somewhat understudied in recent decades (Hertel et al., 2016).

## 5 Conclusion

Given the importance of the livestock sector to human and natural systems we examined how factors of demand may influence livestock-derived food demand in the future. We used a global economic model to simulate scenarios for changes in human population, income, and the income elasticity of demand. Our study has three main conclusions. First, under mid-range trends in population and income and using our reference case elasticities, livestock-derived food demand (for protein) is expected to increase globally by 14% per person and by 38% in total between 2020 and 2050. Demand growth is expected to be fastest in South Asia and sub-Saharan Africa. Second, given the effect of rising incomes and falling prices on demand, substantial reductions in the income elasticity of demand for red meat would be needed to see per person demand in high-income countries fall in 2050 relative to 2020. Prices play an important role in determining demand and the result of a decline in red meat demand in high-income countries is strongly related to rising consumer prices, as projected by our scenario-based modeling. Many factors could lead to a decline in the income elasticity of demand. In our study, however, we focused on the consequences of these declines for demand. We did not explore the pathways that could lead to these declines. Third, global reductions in the income elasticity of demand for red meat can have seemingly counterintuitive results. The demand effect of changes in income or the income elasticity of demand differs based on how sensitive consumers are to price changes and their initial income elasticity of demand and income levels. Lower income growth can lead to a slowing of demand growth in some regions of the world, but this can then reduce global prices and lead to demand increases for consumers who are more price sensitive and less income sensitive. Our study highlights the importance of interactions among demand factors if examining the future of demand for livestock-derived foods. These interactions are important because if the income elasticity of demand changes adjustments may be needed elsewhere in the global food system to assure that any price changes are coherent with the objectives of the global food system.

## Supplementary Material

**Appendix A. Supplementary data**

Supplementary Information (SI) to this study are online at https://doi.org/10.1016/j.gloenvcha.2021.102343. This includes SI Section 1 to SI Section 3, Fig. SI.1 to Fig. SI.9, and Table SI.1 to Table SI.6.

Supplementary information

## Figures and Tables

**Table 1:** Summary of simulation scenarios showing income trajectories, income, and population.

Scenario group	Change in income elasticity trajectory relative to reference case elasticities	SSP	Annual change in per person income 2020 to 2050	Annual change in total population 2020 to 2050
Reference case	No change	SSP1	3.1%	0.4%
Red meat income elasticities	50% less for red meat in all countries	SSP1	3.1%	0.4%
Red meat income elasticities	100% less for red meat in all countries	SSP1	3.1%	0.4%
Red meat income elasticities	50% less for red meat in high-income countries only	SSP1	3.1%	0.4%
Red meat income elasticities	100% less for red meat in high-income countries only	SSP1	3.1%	0.4%
Reference case	No change	SSP2	2.1%	0.6%
Red meat income elasticities	50% less for red meat in all countries	SSP2	2.1%	0.6%
Red meat income elasticities	100% less for red meat in all countries	SSP2	2.1%	0.6%
Red meat income elasticities	50% less for red meat in high-income countries only	SSP2	2.1%	0.6%
Red meat income elasticities	100% less for red meat in high-income countries only	SSP2	2.1%	0.6%
Reference case	No change	SSP3	1.0%	0.9%
Red meat income elasticities	50% less for red meat in all countries	SSP3	1.0%	0.9%
Red meat income elasticities	100% less for red meat in all countries	SSP3	1.0%	0.9%
Red meat income elasticities	50% less for red meat in high-income countries only	SSP3	1.0%	0.9%
Red meat income elasticities	100% less for red meat in high-income countries only	SSP3	1.0%	0.9%

Notes: For the red meat income elasticities scenarios, the income elasticity of demand for each of the three red meats (beef, sheep, pork) linearly declines so that in 2050 its values are either 50% or 100% less than in the reference case. All countries in column 2 refers to all countries in the world and high-income countries are based on the World Bank’s 2020 Country Group classification. SSP is shared socioeconomic pathway. Column 3 indicates which SSP narrative is used for income and population data. Percent changes are based on compound annual growth rate and are the global value rounded to one decimal place, with inter-region and intra-region variability in the ranges existing (Fig. SI.4).

**Table 2:** Annual per person demand for livestock-derived food by region and year (historical and projected).

Food	Indicator (demand in kg, % change is annual)	World	East Asia & Pacific	Europe	Former Soviet Union	Latin America & Caribbean	Middle East & North Africa	North America	South Asia	sub-Saharan Africa
Beef	Demand in 1980	10.6	1.9	21.8	26.2	22.3	5.4	46.6	2.6	7.5
Beef	Demand in 2010	9.5	5.3	16.2	16.2	25.0	8.1	37.8	2.4	5.7
Beef	Demand in 2020	10.9	8.2	17.0	17.7	26.2	8.0	43.1	3.3	6.6
Beef	Demand in 2050	13.1	10.4	18.5	19.3	28.4	11.0	42.6	5.4	11.2
Beef	% change 1980 to 2010	−0.4	3.4	−1.0	−1.6	0.4	1.4	−0.7	−0.3	−0.9
Beef	% change 2020 to 2050	0.6	0.8	0.3	0.3	0.3	1.1	0.0	1.7	1.8
Sheep	Demand in 1980	1.6	0.7	3.0	3.6	1.0	5.1	0.7	1.0	2.5
Sheep	Demand in 2010	1.9	2.2	2.3	2.7	0.8	3.9	0.5	1.0	2.7
Sheep	Demand in 2020	2.5	3.2	2.5	3.0	0.8	5.0	0.6	1.4	2.9
Sheep	Demand in 2050	3.5	3.7	3.0	4.4	1.1	7.0	0.6	2.5	5.1
Sheep	% change 1980 to 2010	0.6	3.8	−0.9	−1.0	−0.5	−0.9	−1.2	−0.1	0.3
Sheep	% change 2020 to 2050	1.2	0.5	0.6	1.3	0.8	1.1	0.2	2.0	1.9
Pork	Demand in 1980	11.9	10.1	38.5	20.6	8.9	0.1	33.1	0.3	0.9
Pork	Demand in 2010	15.6	28.0	39.6	16.6	11.0	0.1	27.6	0.2	1.6
Pork	Demand in 2020	15.3	31.4	38.3	13.2	10.3	0.1	28.1	0.4	1.8
Pork	Demand in 2050	14.2	33.4	38.8	14.7	11.4	0.1	27.6	0.5	3.6
Pork	% change 1980 to 2010	0.9	3.5	0.1	−0.7	0.7	−0.1	−0.6	−0.6	1.9
Pork	% change 2020 to 2050	−0.2	0.2	0.0	0.4	0.4	0.1	−0.1	0.8	2.4
Poultry	Demand in 1980	5.8	2.8	13.4	8.7	8.4	6.9	26.0	0.3	2.1
Poultry	Demand in 2010	14.1	13.0	21.0	18.1	30.6	20.1	49.5	2.1	4.7
Poultry	Demand in 2020	14.1	15.6	21.1	17.1	28.4	18.6	46.7	2.9	3.8
Poultry	Demand in 2050	18.3	22.3	24.2	21.3	35.7	24.3	49.6	8.9	6.1
Poultry	% change 1980 to 2010	3.0	5.2	1.5	2.5	4.4	3.6	2.2	6.7	2.6
Poultry	% change 2020 to 2050	0.9	1.2	0.5	0.7	0.8	0.9	0.2	3.8	1.6
Eggs	Demand in 1980	5.6	4.0	13.5	13.5	6.2	3.9	15.3	0.8	1.7
Eggs	Demand in 2010	8.9	14.4	11.9	12.8	10.3	5.7	13.9	2.3	1.9
Eggs	Demand in 2020	8.8	15.4	11.6	11.8	9.5	6.4	14.0	2.9	1.8
Eggs	Demand in 2050	8.9	16.9	11.5	12.1	10.3	7.3	13.6	4.2	2.7
Eggs	% change 1980 to 2010	1.6	4.4	−0.4	−0.2	1.7	1.2	−0.3	3.6	0.5
Eggs	% change 2020 to 2050	0.0	0.3	0.0	0.1	0.3	0.4	−0.1	1.3	1.3
Milk	Demand in 1980	77.0	12.2	224.0	171.4	102.0	87.3	235.3	41.4	34.3
Milk	Demand in 2010	88.8	31.6	236.2	168.9	120.0	86.1	246.8	83.4	39.1
Milk	Demand in 2020	93.0	49.4	238.8	162.1	116.5	81.6	255.5	88.7	35.9
Milk	Demand in 2050	102.6	77.0	243.8	161.7	130.3	83.6	253.9	100.1	41.6
Milk	% change 1980 to 2010	0.5	3.2	0.2	0.0	0.5	0.0	0.2	2.4	0.4
Milk	% change 2020 to 2050	0.3	1.5	0.1	0.0	0.4	0.1	0.0	0.4	0.5

Notes: 1980 and 2010 are historical data from Food Balance Sheets (FAO, 2020). 2020 and 2050 are projected data simulated using income and population from shared socioeconomic pathway 2 and the reference case elasticities. Sheep includes sheep and goat. Percent change is based on compound annual growth rate.

**Table 3:** Annual per person demand for red meat protein in 2020 and percent change in demand (2020 to 2050) under changes in red meat income elasticities by SSP.

SSP	Country group		Scenario for change in income elasticity trajectory				
			Reference case (no change)	50% lower all countries	100% lower all countries	50% lower HIC only	100% lower HIC only
		Reference case average per person demand in 2020 (kg)	Percent change in per person demand 2020 to 2050				
SSP1	All	4.6	22.3	5.1	−7.0	21.4	20.6
SSP2	All	4.6	10.1	−2.0	−11.2	9.4	8.7
SSP3	All	4.5	−2.7	−10.6	−17.1	−3.2	−3.6
SSP1	HIC	9.0	3.9	7.8	10.6	−0.5	−4.3
SSP2	HIC	9.0	4.3	6.8	8.9	0.5	−2.8
SSP3	HIC	9.0	4.7	6.1	7.4	1.4	−1.5
SSP1	LMIC	3.8	30.3	3.6	−15.5	30.9	31.4
SSP2	LMIC	3.8	14.5	−3.7	−17.9	14.9	15.3
SSP3	LMIC	3.7	0.8	−10.4	−19.7	1.1	1.4

Notes: Data in columns 4 to 8 are percent changes between 2020 and 2050 in each of the five scenarios listed in the second row. Demand is in protein and is summed over beef, sheep, and pork. Projected data simulated using income and population from three shared socioeconomic pathways (SSP). Average in 2020 reference case is demand from all countries in a country group divided by total population from all countries in a country group. All = all countries in the world, HIC = high-income countries, LMIC = low- and middle-income countries. SI Section 3 describes an additional scenario where red meat becomes an inferior commodity in high-income countries only, with Table SI.6 reporting the results of the additional scenario.

## Data Availability

The data and scripts for this study are available at https://doi.org/10.7910/DVN/ZPWQBB.
