# Supplementary material for: Income, consumer preferences, and the future of livestock-derived food demand
Source: Glob Environ Change. Author manuscript; Available in PMC 2021 Dec 1. (PMC7612057; doi:10.1016/j.gloenvcha.2021.102343)
Supplement: Supplementary information [file EMS140038-supplement-Supplementary_information.docx]

# Supplementary Information (SI)

This file contains Supplementary Information for the study titled “Income, consumer preferences, and the future of livestock-derived food demand” <https://doi.org/10.1016/j.gloenvcha.2021.102343>

## SI Section 1: modeling details

### IMPACT model demand, supply, and price details

This section reports how we simulated demand and supply (production) and prices for livestock-derived foods. The text and equations in this section are an almost verbatim reporting of the model description in Robinson *et al.* (2015), who provide additional information on the model’s technical details. At the end of every year the world’s demand for a commodity (*c*) equals the world’s supply of that commodity. To ensure global demand equals global supply the sum of net trade over all countries in the world must equal zero. In each country (*cty*) Eq. S1 holds:

$$\begin{matrix} NT_{\text{c,cty}}=QSUP_{\text{c,cty}}-QD_{\text{c,cty}}-QSt_{\text{c,cty}}\text{ (S1)} \\ NT=\text{Net trade} \\ QSUP\text{ = Production (supply)} \\ QD\text{ = Final demand} \\ QSt=\text{Change in stocks} \end{matrix}$$

Eq. S2 simulates final demand (*QD*). This final demand for a commodity is the sum of household food demand (*QH*), intermediate demand from the agricultural-processing sector (*QInterm*), feed demand from the livestock sector (*QL*), intermediate demand for biofuels as a feedstock (*QBF*), and other demand (*QOTH*). This other demand summarizes all other demands for agricultural products from sectors outside of the focus of IMPACT, including for industrial processes (e.g. other uses in FAOSTAT), seed production, and losses along the value chain between farm-gate and final purchase by consumers.

$$\begin{matrix} QD_{\text{c,cty}}=QH_{\text{c,cty}}+QInterm_{\text{c,cty}}+QL_{\text{c,cty}}+QBF_{\text{c,cty}}+QOTH_{\text{c,cty}}\text{ (S2)} \\ QD=\text{Total commodity demand} \\ QH=\text{Total household food demand} \\ QInterm=\text{Intermediate demand from agricultural-processing sector} \\ QL=\text{Feed demand from livestock sector} \\ QBF=\text{Intermediate demand for biofuel feedstock} \\ QOth=\text{All other demand} \end{matrix}$$

Eq. S3 simulates total household quantity demanded by consumers (*QH*) for agricultural commodities (including livestock-derived food commodities) in a country in a year, and *cc* is a different commodity to *c*. In Eq. S2 household demand and consumer prices are endogenous variables, and model parameters include per person income, human population, consumer support estimates, the income elasticity of demand, and the price elasticity of demand. Solution values for variables and parameter values are carried forward between years and are used in solving the current year. For example, if Eq. S3 is solving for demand in the year 2021 then initial household demand or initial per person income or initial population are the values from 2020. Eq. S3 reflects consumer food demand and does not include final non-food demand for agricultural products (e.g. pet food or industrial products using agricultural inputs), which are included in other demand described in Eq. S2. Although food losses, as reported in the Supply Utilization Tables in FAOSTAT’s Food Balance Sheets, are also included in other demand in IMPACT, consumer food waste is not included. As such food consumption is always less than food demand. Nevertheless, changes in food demand are a useful proxy for changes in food consumption, as waste fractions are relatively stable.

The demand function in Eq. S3 relates to the neoclassical theory of consumer behavior that assumes consumers are rational. This theory has typically been described as having three steps: (1) consumer preferences, (2) budget constraints, and (3) consumer choices (Pindyck and Rubinfeld, 2015). Consumers allocate their income to buy a bundle (or market basket) of goods. The bundle is a list with specific quantities of one or more goods. It is assumed that consumers have a set of preferences that they use to guide them in choosing between bundles. Underlying the theory is the belief that consumers can compare among bundles and decide which bundle gives them the greatest pleasure. The preferences can be summarized by assigning a numerical value to each bundle to reflect the ranking of all available bundles. The word utility has been applied to this set of numerical values to reflect the relative ranking of the bundles. And a utility function provides the relationship between utility and every possible bundle. Knowing consumer preferences is the first step in understanding consumer choices, as in the neoclassical theory of consumer behavior consumers maximize utility subject to constraints. An important constraint is the budget constraint, and a common hypothesis is that a rational consumer will always choose a most preferred bundle from a set of affordable alternatives (Varian, 1992). The amount of goods that can be purchased at given prices is limited by income. Specifically related to Eq. S3, prices and income are directly in the equation. Consumer prices (PC) are endogenous (this section and Section 2) and income is exogenous to our model and is taken from the SSP database (Section 2). For consumer preferences, we assume that changes in the income elasticity of demand (an exogenous parameter in our model) are a partial proxy for changes in consumer preferences. For example, take a scenario where per person consumption (for a commodity-country-year combination) is 10 kg and if a 1% increase in income leads to a demand being 10.03 kg this means that the income elasticity of demand is 0.3. Then take a counterfactual to this scenario, if income increased by 1% and the new level of demand was 10.02 kg (instead of 10.03 kg) this means that the income elasticity of demand is 0.2. We assume that the change in elasticity from 0.3 to 0.2 between the scenarios was because of a change in consumer preferences that then changed how the consumer allocated their income to the consumption bundle given prevailing prices.

$$\begin{matrix} QH_{\text{c,cty}}=QHInt_{\text{c,cty}}\times\left(\frac{pcGDP_{\text{cty}}}{pcGDP0_{\text{cty}}}\right)^{Inc\varepsilon}\times\left(\frac{(1-CSE_{\text{c,cty}})\times PC_{\text{c,cty}}}{(1-CSE0_{\text{c,cty}})\times PC0_{\text{c,cty}}}\right)^{HF\varepsilon}\times\text{ (S3)} \\ \prod_{cc\neq c} \left(\frac{(1-CSE_{\text{cc,cty}})\times PC_{\text{cc,cty}}}{(1-CSE0_{\text{cc,cty}})\times PC0_{\text{cc,cty}}}\right)^{HF\varepsilon}\times\frac{PopH_{\text{cty}}}{PopH0_{cty}} \\ \\ QH=\text{Household food demand (thousand metric tons)} \\ QHInt=\text{Initial household food demand (thousand metric tons)} \\ pcGDP=\text{Per person income (billion constant 2005 US dollars, gross domestic product)} \\ pcGDP0=\text{Initial per person income (billion constant 2005 US dollars, gross domestic product)} \\ CSE=\text{Consumer support estimate (percentage (ad valorem) of the price)} \\ CSE0=\text{Initial consumer support estimate (percentage (ad valorem) of the price)} \\ PC=\text{Consumer price (constant 2005 US dollars per metric ton)} \\ PC0=\text{Initial consumer price (constant 2005 US dollars per metric ton)} \\ PopH=\text{Population (million people)} \\ PopH0=\text{Initial household population (million people)} \\ Inc\varepsilon=\text{Income elasticity of demand (unitless)} \\ HF\varepsilon=\text{Price elasticity of demand (unitless)} \\ \left(\frac{(1-CSE)\times PC}{(1-CSE0)\times PC0}\right)^{HF\varepsilon}=\text{Own-price response} \\ \prod_{cc\neq c} \left(\frac{(1-CSE)\times PC}{(1-CSE0)\times PC0}\right)^{HF\varepsilon}=\text{Cross-price response} \end{matrix}$$

Eq. S4 simulates the total number of animals from livestock production activities (*j*) in a subnational food production unit (*fpu*) in a livestock system (*livsys*) in a year. Eq. S4 is only for the production activities that produce commodities beef, sheep, pork, poultry, eggs, and milk. All countries contain at least one *fpu*. Animal numbers (*Animals*) in the current simulation year are a function of the previous year’s animal numbers (*AnimalInt*), an exogenous trend over time reflecting growth in animal numbers (*AnmlInt2*), producer prices (*PP*), and the prices of feed grain crops (*PC*). Initial animal numbers in the first year of the model are set based on FAOSTAT animal numbers.

$$\begin{matrix} Animals_{\text{j,fpu,livsys}}=AnimalInt_{\text{j,fpu,livsys}}\times AnimalInt2_{\text{j,fpu,livsys}}\times\left(\frac{PP_{\text{j,cty}}}{PP0_{\text{j,cty}}}\right)^{AN\varepsilon}\times\prod_{\text{cfeeds}} \left(\frac{PC_{\text{c,cty}}}{PC0_{\text{c,cty}}}\right)^{Feed\varepsilon}\text{ (S4)} \\ Animals=\text{Number of animals (000 head of animals)} \\ AnimalInt=\text{Animal intercept (initial number of animals, 000 head of animals)} \\ AnimalInt2=\text{Exogenous growth of animal numbers} \\ PP=\text{Producer prices (constant 2005 US dollars per metric ton)} \\ PP0=\text{Initial producer prices (constant 2005 US dollars per metric ton)} \\ AN\varepsilon=\text{Price elasticity of supply with respect to changes in livestock price (unitless)} \\ PC=\text{Consumer prices (constant 2005 US dollars per metric ton)} \\ PC0=\text{Initial consumer prices (constant 2005 US dollars per metric ton)} \\ Feed\varepsilon=\text{Price elasticity of supply with respect to changes in feed prices (unitless)} \\ cfeeds=\text{Feed commodities demanded by animals} \end{matrix}$$

Livestock yields are the quantity of output per animal (meat, milk, or eggs) and these yields are determined through an exogenous growth parameter (Eq. S5). The exogenous growth parameter is based on intrinsic productivity growth rates and is related to improved animals and management practices. The intrinsic productivity growth rates are a series of commodity- and country-specific assumptions on non-price (exogenous) productivity growth (through the year 2050). The intrinsic productivity growth rates attempt to summarize historical trends on productivity increases and opinion on the future returns of agricultural research and development. These intrinsic productivity growth rates, to varying degrees, build on productivity trends observed during the past 60 years and are also informed by collaborative knowledge gained under the Global Futures and Strategic Foresight project regarding expected gains in productivity in the future (based on expectations about future private and public sector research and extension efforts). These intrinsic productivity growth rates are our assumptions. The intrinsic productivity growth rates for each of the six livestock-derived foods vary between and within regions and are typically higher for meat than milk and eggs (Fig. SI.10). Existing studies report additional details on the intrinsic productivity growth rates (Wiebe *et al.*, 2015; Brooks and Place, 2019).

$$\begin{matrix} AnimalYield_{\text{j,fpu,livsys}}=AnimalYieldInt_{\text{j,fpu,livsys}}\times AnimalYieldInt2_{\text{j,fpu,livsys}}\text{ (S5)} \\ AnimalYield=\text{Animal yields} \\ AnimalYieldInt=\text{Initial animal yields} \\ AnimalYieldInt2=\text{Exogenous yield growth} \end{matrix}$$

Total production from each activity in a country (*QS*) is calculated by multiplying the number of animals by the yield per animal and summing across *FPU* and livestock system within a country (Eq. S6). Eq. S7 converts total national production to commodity supply. The parameter *JCRatio* maps from the activity output to commodities. Usually, each activity produces a matched commodity. The specification, however, is general. Several activities can produce the same commodity, or a single activity can produce more than one commodity. The units of *j* agree with the units of the main commodity produced by the activity so that the *JCRatio* for this mapped commodity always equals 1.

$$QS_{\text{j,cty}}=\sum_{\text{fpu,livsys}} \left(Animals_{\text{j,fpu,livsys}}\times AnimalYield_{\text{j,fpu,livsys}}\right)\text{ (S6)}$$

Commodity prices are endogenous, and prices are in constant 2005 US dollars. The model has three separate, but linked, markets to jointly determine prices: (1) a farm market, where producers sell their output to purchasers at producer prices; (2) a national market, where the purchasers then take the commodity, incurring any taxes or subsidies and trade or transportation costs; and (3) the international market where exports are sold, and imports are purchased at world market prices. International trade incurs marketing margin costs and any taxes, subsidies, or tariffs. Producer- and consumer-support estimates and marketing margins are expressed as percentages (ad valorem) of the world price. To calculate producer prices (PP) the appropriate wedges are applied to the domestic consumer prices (PC) and represent the markup observed in domestic markets from the price producers receive (Eq. S8). The producer price of an activity is the weighted sum of the prices of the commodities associated with that activity.

$$\begin{matrix} PP_{\text{j,cty}}\times\left(1+MMJ_{\text{j,cty}}\right)=\left(1+PSE_{\text{j,cty}}\right)\times\sum_{\text{c}} JCRatio_{\text{j,c,cty}}\times PC_{\text{c,cty}}\text{ (S8)} \\ PP=\text{Producer price} \\ MMJ=\text{Farm(factory)-gate to domestic market Marketing Margin (MM)} \\ PSE=\text{Producer support estimate, ad valorem component} \\ JCRatio\text{ = mapping from activities (j) to commodities (c)} \end{matrix}$$

How consumer prices are determined depends on if the commodity is tradable or non-tradable on international markets. Traded commodity prices are determined in international markets. Nontraded commodities are those commodities whose prices are determined in national markets, without direct links to international markets. Examples include sugarcane, sugar beets, and grass, where all demand is intermediate demand from domestic sectors (sugar processing and livestock). These commodity prices are determined endogenously and ensure that domestic supply (QSUP) equals domestic demand (QD).

Nontraded commodities are indirectly linked to world markets through the demand for final products (that is, sugar), and potential substitution from tradable commodities (that is, grass and other feeds). The tradability of a commodity is determined endogenously. As the model includes price wedges between domestic and international markets, the prices of exports received by producers and of imports paid by consumers can be modeled in separate equations (Eq. S9 and Eq. S10).

$$\begin{matrix} PM_{\text{c,cty}}=PW_{\text{c}}\times EXR_{\text{cty}}\times\left(1+TM_{\text{c,cty}}\right)\times\left(1+MMM_{\text{c,cty}}\right)\text{ (S9)} \\ PE_{\text{c,cty}}=PW_{\text{c}}\times EXR_{\text{cty}}\times\left(1-TE_{\text{c,cty}}\right)\times\left(1-MME_{\text{c,cty}}\right)\text{ (S10)} \\ \text{Imports if }PC_{\text{c,cty}}\leq PM_{\text{c,cty}} \\ \text{Exports if }PC_{\text{c,cty}}\geq PE_{\text{c,cty}} \\ \text{Domestically traded if }PE_{\text{c,cty}}\leq PC_{\text{c,cty}}\leq PM_{\text{c,cty}}\text{ } \\ PM=\text{Import Price} \\ PE=\text{Export Price} \\ PW=\text{World Price} \\ EXR=\text{Exchange Rate} \\ TM=\text{Import tariff (ad valorem)} \\ TE=\text{Export tax (ad valorem)} \\ MMM=\text{Marketing margin for importing to domestic market} \\ MME=\text{Marketing margin for exporting to international market} \end{matrix}$$

If the equilibrium domestic price falls between the floor price of exports and the ceiling price of imports, then there will be no international trade. If the equilibrium domestic price either falls to the export price or rises to the import price, the regime will change and clear the market through international trade. Imports occur if the domestic import price equals the consumer price (i.e., global prices are lower than domestic prices), and exports start if domestic prices equal export prices (i.e., domestic prices are greater than global prices). For purely tradable goods, the domestic consumer price is set to the import price and the export price equation is never used.

### GLOBE to IMPACT link

We simulated 15 scenarios in our study using a combination of income and population from three SSPs and for each SSP there were five income elasticity scenarios (reference case and four red meat income elasticities). All 15 scenarios were simulated using Representative Concentration Pathway (RCP) 6.0 (Moss *et al.*, 2010) in the HadGEM2-ES general circulation model (GCM) (Jones *et al.*, 2011) and with CO_2_ fertilization of 379 parts per million. The narratives of each SSP by themselves assume no climate change or climate impacts, and no new climate policies (O’Neill et al., 2014). Therefore, we updated the income data used in all our scenarios to account for the climate change induced changes in agricultural production and its economywide effects on income. We did this updating through an iterative procedure between IMPACT and GLOBE (Willenbockel et al., 2018; Delzeit et al., 2020). The size of the effect of climate change on per person income is closely correlated with the share of agriculture in gross domestic product, for example, higher in South Asia and sub-Saharan Africa and lower in high-income countries (Fig. SI.11).

GLOBE is a dynamic general equilibrium model of the world that is calibrated to a Social Accounting Matrix representation of the Global Trade Analysis Project database (Aguiar et al., 2019). We augment our partial equilibrium multimarket economic model to include the macroeconomic impacts of climate change or changes in income elasticities on income and update the income in each SSP accordingly. Changes in agricultural productivity induced by climate change as included in IMPACT are passed to GLOBE as factor productivity shifters that generate equivalent impacts on agricultural producer prices. Price changes lead to knock-on effects for non-agricultural sectors, ultimately leading to changes in incomes. For income elasticities, a decline in an income elasticity for a livestock-derived food in a country that is a net importer of that livestock-derived food will mean fewer imports from other countries of that livestock-derived food, and consequently, the terms of trade will improve, and income will increase. Conversely, the terms of trade of countries that export less will see a deterioration and income will decline, and there will be ripple-on-effects across the board. The steps taken were as follows:

1. The 15 scenarios in Table 1 were run in IMPACT using income levels from the SSP database. The SSPs are reference pathways that assume no climate change or climate impacts, and no new climate policies (O’Neill et al., 2014).
2. GLOBE was run to calculate in each of the 15 scenarios what the change in income from the SSP database income levels was given climate change and changes in elasticities (embedded in each scenario). This process generated a GLOBE-adjusted income trajectory for all countries and years. The GLOBE-adjusted income accounts for the change in income because of climate change induced productivity shocks in agriculture and change in income elasticities.
3. The 15 scenarios in Table 1 were then rerun in IMPACT by incorporating these income dynamics (i.e., using the GLOBE-adjusted income relevant to each of the 15 scenarios) into its demand system, with income being a parameter in each scenario. All results reported in our study are based on GLOBE-adjusted incomes.

This coupling of IMPACT and GLOBE has been applied in existing modeling studies (Ringler *et al.*, 2016; Willenbockel *et al.*, 2018; Mason-D'Croz *et al.*, 2019).

### Uncertainty

Risk is commonly defined as the case where the distribution of outcomes is known either *a priori* or statistically through experience, and uncertainty as the case where probabilities cannot be quantified (Knight, 1921). The red meat income elasticities scenarios are unrelated to the uncertainty of the income elasticity of demand; they are what-if scenarios related to how changes in the income elasticity of demand may alter livestock-derived food demand. We examined how a range of combinations for population, income, and the income elasticity of demand for red meat would affect livestock-derived food demand, but we made no assumptions on the probability of a different trajectory for population and income being realized, or on the probability of what changes in the income elasticity of demand may occur or their magnitude. There exists uncertainty in how demand may evolve over time, including the role of the prices and the role of meat substitutes such as *in vitro* meat (Thornton, 2010). Other sources of uncertainty include major largely unanticipated events, such as global economic crises or health pandemics. These events occurred throughout the 20^th^ century (Just, 2001) and continue to occur. The consequences of these events can be devastating and can lead to, for example, depressed economic growth and more regionalization at the global scale, and this situation is more aligned with an SSP3 trajectory for income than the mid-range SSP2 trajectory for income. Improvements in data and analytical methods that take either a frequentist or subjectivist view on the probability of different trends in factors of demand caused by major unanticipated events, among other factors, may help improve our ability to project demand for livestock-derived food.

Another issue related to uncertainty for models that examine long-term projections is how to compare historical data with projected data. Although it is often desirable for a model to be able to mimic some features of historical data (Cooley, 1997), we are also mindful that future demand may be controlled by different processes than existed in the past. The degree to which the future may repeat itself is unknown. A desire for simulated data to mimic observed data from the past should not be overstated because it risks over training (or over calibrating) a model to a situation in the past that may not occur again in the future, and this is because past processes might not necessarily be the processes driving the future (Verburg *et al.*, 2006; Reilly and Willenbockel, 2010; Uthes *et al.*, 2010). Global economic models are typically designed for use in the analyses of scenarios outside of the domain of historical data. In our study we projected demand under a range of scenarios, and this is distinct from forecasting analysis in that our objective was not to predict the most likely outcome (usually extrapolating from historical experience).

### Global economic model details

The increases in livestock calorie demand between 2005 and 2050 reported across 11 global economic models ranged from 12 to 140% for per person demand and from 61 to 242% for total demand (Valin *et al.*, 2014). This range occurs because of differences in how each model specifies its demand system and its income and price elasticities, among others (Valin *et al.*, 2014; von Lampe *et al.*, 2014). For example, elasticities in the 11 models are from a range of sources including USDA (1998), the Global Trade Analysis Project (Hertel *et al.*, 2014), FAO projections, and some price elasticities are endogenously determined, such as in the models that use a Linear Expenditure System for demand. The type of demand system used in IMPACT is also used in the global economic models of Global Change Assessment Model and Global Biosphere Management Model (Valin *et al.*, 2014). Existing studies provide additional details on differences between the 11 global economic models (Valin *et al.*, 2014; von Lampe *et al.*, 2014).

Our projected increases in demand (Results Section 3) are based on IMPACT version three and these projected increases are less than those projected increases reported in IMPACT version two (Valin *et al.*, 2014). These differences primarily arise because IMPACT version three (relative to IMPACT version two) contains 1) different income and population data, from IIASA and OECD (Section 2.2.2) not the United Nations and the World Bank, 2) updated demand elasticities based on periodic updating of elasticities (described in Methods Section 2), and calorie content of each livestock-derived food was taken from the Global Expanded Nutrient Supply (GENuS) model (Smith *et al.*, 2016) not FAOSTAT.

### GENuS data

This section describes briefly the GENuS data (Smith *et al.*, 2016), and the text in this section heavily relies on existing descriptions of GENuS, such as Beach *et al.* (2019). GENuS begins by estimating the per person availability of 225 foods in the diet by using a combination of the FAO Food Balance Sheets—which provide estimates on the per capita availability of roughly 100 foods in most countries globally—and additional production and trade data provided by the FAO which were used to approximately replicate the Food Balance Sheets methodology (total quantity of food production and imports minus exports and uses other than human consumption) and increase the number of described foods to 221. Non-edible portions of each food (e.g., bones, peels, seeds), were removed at this stage, leaving the edible per capita supply of each food as the intermediate output. Twenty-three countries without sufficient data to allow for the expansion of foods described were removed from the dataset. Per capita food supplies for each country were then paired with one of six regional food composition tables — USA, West Africa, India, Northeast Asia, Southeast Asia, and Latin America — to infer the nutrient supply across 23 nutrients by food and country. All foods in each table that were suitable matches for a GENuS food category were included in the analysis, and Monte Carlo simulations (N=1,000) were to estimate the uncertainty inherent in the estimates of the nutrients provided by each country’s diet. The median and 95% uncertainty intervals of the Monte Carlo simulations were reported and included as inputs in the analysis of Smith *et al.* (2016). Smith *et al.* (2016) provide a comparison of GENuS estimates of nutrient supplies in the USA with estimates from the USDA Nutrient Database.

## SI Section 2: Simpson’s paradox

Our results suggest that average per person pork demand globally under SSP2 is lower in 2050 than in 2020, but per person demand for pork under SSP2 in seven of the eight regions increased between 2020 and 2050 (Table 2). In 2050 a larger share of the world’s population is expected to live in regions with demand below the global average (i.e., living in South Asia or sub-Saharan Africa not Europe or North America), compared to the population share in 2020 living in those same regions. Regions with faster population growth generally have lower per person demand than in regions with slower population growth. This means that the global average for per person demand falls, even though actual demand in most regions rises between 2020 and 2050. This Simpson’s paradox has been described for food consumption data in existing studies (Alexandratos, 1999).

## SI Section 3: normal versus inferior goods

To extend the red meat income elasticities scenarios beyond the range reported in Table 3, we ran an additional scenario where we asked the question what would happen to demand for red meat if in IMPACT the red meat commodities of beef, sheep, and pork switched from being classified a normal good to an inferior good by the year 2050. In this scenario we adjusted the downward time trend for red meat income elasticities of demand so that in 2050 all the red meat income elasticities of demand in high-income countries were −0.2. Most livestock-derived foods have positive income elasticities in all years (Fig. SI.2) (i.e., they are classified as a normal good) and the change to a negative income elasticity of demand by 2050 in the additional scenario means these livestock-derived foods switch over time from being a normal good to an inferior good. Here the word “inferior” means that when incomes rise demand falls (Pindyck and Rubinfeld, 2015). The results for the additional scenario in Table SI.6 show that if income elasticities for red meat in high-income countries decline faster (relative to the 100% decline scenario in the penultimate column) then there is a commensurate decline in per person demand. For example, if elasticities are zero in 2050 (the typical case in the penultimate column in Table SI.6) then per person demand in high-income countries is 2.8% less in 2050 than in 2020, but if elasticities in 2050 are even lower than zero and set at −0.2 then per person demand in high-income countries declines by even more, i.e., 10.1% less in 2050 than in 2020.

## SI tables

**Table SI.1**

Average (and range) grams of protein per 100 grams of each livestock-derived food by region

| Region | Beef | Sheep | Pork | Poultry | Eggs | Milk |
| --- | --- | --- | --- | --- | --- | --- |
| EAP | 17.8 (17.3–19.4) | 17.3 (16.5–18.9) | 13.9 (13.2–16.5) | 18.8 (18.6–19.4) | 12.7 (12.6–13.3) | 3.1 (3.0–3.4) |
| EUR | 17.3 (17.3–17.3) | 16.9 (16.9–16.9) | 13.9 (13.9–13.9) | 18.6 (18.6–18.6) | 12.6 (12.6–12.6) | 3.2 (3.1–3.2) |
| FSU | 17.3 (17.3–17.3) | 16.9 (16.9–16.9) | 13.9 (13.9–13.9) | 18.6 (18.6–18.6) | 12.6 (12.6–12.6) | 3.2 (3.1–3.2) |
| LAC | 19.3 (17.3–19.4) | 18.8 (16.9–18.9) | 19.1 (13.9–19.5) | 18.0 (18.0–18.6) | 12.5 (12.5–12.6) | 3.3 (3.2–3.3) |
| MEN | 17.4 (17.3–18.4) | 16.8 (16.5–16.9) | 14.5 (13.9–18.7) | 18.7 (18.6–19.5) | 12.6 (12.6–12.6) | 3.2 (3.1–3.4) |
| NAM | 17.3 (17.3–17.3) | 16.9 (16.9–16.9) | 13.9 (13.9–13.9) | 18.6 (18.6–18.6) | 12.6 (12.6–12.6) | 3.2 (3.2–3.2) |
| SAS | 19.1 (17.3–19.4) | 18.6 (16.9–18.9) | 17.9 (13.2–19.0) | 19.4 (18.6–19.9) | 13.1 (12.6–13.3) | 3.3 (3.1–3.4) |
| SSA | 18.4 (18.4–18.4) | 16.4 (16.4–16.5) | 16.7 (13.9–16.8) | 19.5 (19.5–19.5) | 12.6 (12.6–12.6) | 3.4 (3.4–3.4) |

Notes: Data derived from the Global Expanded Nutrient Supply (GENuS) Model (Smith *et al.*, 2016) for consistent values in 2011. SI Section 1 provides a summary of the GENuS model. EAP = East Asia & Pacific , EUR = Europe, FSU = Former Soviet Union, LAC = Latin America & Caribbean, MEN = Middle East & North Africa, NAM = North America, SAS = South Asia, SSA = sub-Saharan Africa.

**Table SI.2**

Descriptive summary (non-inferential statistics) for country-scale annual percent change in per person livestock-derived food demand between 2020 and 2050

| Livestock-derived food | Minimum | Lower quartile | Median | Average | Upper quartile | Maximum |
| --- | --- | --- | --- | --- | --- | --- |
| Beef | −0.29 | 0.29 | 0.90 | 1.07 | 1.46 | 4.92 |
| Sheep | 0.00 | 0.71 | 1.17 | 1.25 | 1.66 | 3.73 |
| Pork | −0.27 | 0.23 | 0.96 | 1.20 | 1.85 | 4.15 |
| Poultry | −0.12 | 0.65 | 1.38 | 1.48 | 2.03 | 5.39 |
| Eggs | −0.91 | 0.02 | 0.10 | 0.34 | 0.38 | 3.27 |
| Milk | −0.15 | 0.15 | 0.93 | 0.86 | 1.46 | 2.71 |

Notes: Projected data simulated using income and population from shared socioeconomic pathway 2 and the reference case elasticities. Data are for all countries in the world. Lower quartile is 25^th^ percentile and upper quartile is 75^th^ percentile. Percent change based on compound annual growth rate.

**Table SI.3**

Total annual demand for livestock-derived food by region and year

|  |  |  |  |  |  |  |  |  |  |  |
| --- | --- | --- | --- | --- | --- | --- | --- | --- | --- | --- |
| Food | Indicator  (demand in million metric tons, % change is annual) | World | East Asia & Pacific | Europe | Former Soviet Union | Latin America & Caribbean | Middle East & North Africa | North America | South Asia | sub-Saharan Africa |
| Beef | Demand in 1980 | 46.5 | 3.0 | 10.6 | 7.0 | 8.0 | 1.2 | 11.9 | 2.3 | 2.6 |
| Beef | Demand in 2010 | 64.5 | 11.8 | 8.7 | 4.5 | 14.8 | 3.4 | 13.1 | 3.8 | 4.4 |
| Beef | Demand in 2020 | 83.5 | 18.7 | 9.4 | 5.0 | 16.9 | 4.3 | 16.1 | 6.1 | 7.1 |
| Beef | Demand in 2050 | 120.6 | 23.5 | 10.6 | 5.3 | 21.1 | 7.9 | 19.2 | 12.8 | 20.1 |
| Beef | % change 1980 to 2010 | 1.1 | 4.7 | −0.6 | −1.4 | 2.1 | 3.6 | 0.3 | 1.7 | 1.8 |
| Beef | % change 2020 to 2050 | 1.2 | 0.8 | 0.4 | 0.2 | 0.7 | 2.0 | 0.6 | 2.5 | 3.5 |
| Sheep | Demand in 1980 | 7.0 | 1.1 | 1.5 | 1.0 | 0.3 | 1.1 | 0.2 | 0.9 | 0.9 |
| Sheep | Demand in 2010 | 12.8 | 4.8 | 1.2 | 0.8 | 0.5 | 1.6 | 0.2 | 1.6 | 2.1 |
| Sheep | Demand in 2020 | 18.7 | 7.3 | 1.4 | 0.8 | 0.5 | 2.7 | 0.2 | 2.6 | 3.1 |
| Sheep | Demand in 2050 | 32.4 | 8.4 | 1.7 | 1.2 | 0.8 | 5.0 | 0.3 | 5.9 | 9.1 |
| Sheep | % change 1980 to 2010 | 2.0 | 5.0 | −0.5 | −0.8 | 1.1 | 1.3 | −0.2 | 1.8 | 3.0 |
| Sheep | % change 2020 to 2050 | 1.8 | 0.5 | 0.7 | 1.2 | 1.3 | 2.1 | 0.8 | 2.8 | 3.6 |
| Pork | Demand in 1980 | 52.0 | 15.7 | 18.7 | 5.5 | 3.2 | 0.0 | 8.4 | 0.3 | 0.3 |
| Pork | Demand in 2010 | 105.7 | 62.0 | 21.3 | 4.7 | 6.5 | 0.0 | 9.6 | 0.4 | 1.2 |
| Pork | Demand in 2020 | 116.5 | 71.9 | 21.2 | 3.7 | 6.6 | 0.0 | 10.5 | 0.7 | 1.9 |
| Pork | Demand in 2050 | 130.4 | 75.5 | 22.4 | 4.1 | 8.5 | 0.1 | 12.4 | 1.1 | 6.4 |
| Pork | % change 1980 to 2010 | 2.4 | 4.7 | 0.4 | −0.5 | 2.4 | 2.1 | 0.4 | 1.3 | 4.7 |
| Pork | % change 2020 to 2050 | 0.4 | 0.2 | 0.2 | 0.3 | 0.8 | 1.1 | 0.6 | 1.7 | 4.1 |
| Poultry | Demand in 1980 | 25.3 | 4.4 | 6.5 | 2.3 | 3.0 | 1.5 | 6.6 | 0.3 | 0.7 |
| Poultry | Demand in 2010 | 95.7 | 28.8 | 11.3 | 5.1 | 18.1 | 8.5 | 17.2 | 3.3 | 3.6 |
| Poultry | Demand in 2020 | 107.5 | 35.8 | 11.7 | 4.8 | 18.3 | 10.0 | 17.4 | 5.4 | 4.1 |
| Poultry | Demand in 2050 | 168.5 | 50.5 | 14.0 | 5.9 | 26.5 | 17.4 | 22.3 | 21.1 | 10.9 |
| Poultry | % change 1980 to 2010 | 4.5 | 6.5 | 1.9 | 2.7 | 6.1 | 6.0 | 3.2 | 8.8 | 5.4 |
| Poultry | % change 2020 to 2050 | 1.5 | 1.1 | 0.6 | 0.7 | 1.2 | 1.9 | 0.8 | 4.7 | 3.3 |
| Eggs | Demand in 1980 | 24.5 | 6.1 | 6.5 | 3.6 | 2.2 | 0.9 | 3.9 | 0.7 | 0.6 |
| Eggs | Demand in 2010 | 60.4 | 32.0 | 6.4 | 3.6 | 6.1 | 2.4 | 4.8 | 3.6 | 1.5 |
| Eggs | Demand in 2020 | 67.1 | 35.2 | 6.4 | 3.3 | 6.1 | 3.5 | 5.2 | 5.3 | 2.0 |
| Eggs | Demand in 2050 | 82.1 | 38.2 | 6.6 | 3.3 | 7.7 | 5.3 | 6.1 | 10.0 | 4.9 |
| Eggs | % change 1980 to 2010 | 3.0 | 5.7 | −0.1 | 0.0 | 3.4 | 3.5 | 0.7 | 5.7 | 3.2 |
| Eggs | % change 2020 to 2050 | 0.7 | 0.3 | 0.1 | 0.0 | 0.7 | 1.4 | 0.5 | 2.1 | 3.0 |
| Milk | Demand in 1980 | 338.1 | 19.0 | 108.6 | 45.5 | 36.7 | 19.0 | 59.9 | 37.4 | 11.9 |
| Milk | Demand in 2010 | 601.4 | 70.0 | 127.2 | 47.4 | 70.9 | 36.2 | 85.5 | 133.9 | 30.2 |
| Milk | Demand in 2020 | 709.0 | 113.1 | 132.0 | 45.6 | 75.0 | 43.8 | 95.4 | 165.1 | 39.0 |
| Milk | Demand in 2050 | 942.5 | 174.2 | 140.7 | 44.8 | 96.6 | 59.8 | 114.3 | 237.6 | 74.6 |
| Milk | % change 1980 to 2010 | 1.9 | 4.5 | 0.5 | 0.1 | 2.2 | 2.2 | 1.2 | 4.3 | 3.1 |
| Milk | % change 2020 to 2050 | 1.0 | 1.4 | 0.2 | −0.1 | 0.8 | 1.0 | 0.6 | 1.2 | 2.2 |

Notes: 1980 and 2010 are historical data from Food Balance Sheets (FAO, 2020). 2020 and 2050 are projected data simulated using income and population from shared socioeconomic pathway 2 and the reference case elasticities. Percent change based on compound annual growth rate.

**Table SI.4**

Annual demand for red meat in 2020 and percent change in demand between 2020 and 2050 under SSP2 for different scenarios for changes in red meat income elasticities

| Country group | Livestock-derived food(s) | Unit | Demand type |  |  | | Scenario for change in income elasticity trajectory | | | | |
| --- | --- | --- | --- | --- | --- | --- | --- | --- | --- | --- | --- |
|  |  |  |  |  |  | Reference case | | 50% lower all countries | 100% lower all countries | 50% lower HIC only | 100% lower HIC only |
|  |  |  |  | Reference case average demand in 2020 |  | Percent change in demand 2020 to 2050 | | | | | |
| All | Beef | Food | Per person | 10.9 |  | 19.9 | | 5.3 | −5.2 | 19.2 | 18.6 |
| HIC | Beef | Food | Per person | 24.8 |  | 6.7 | | 10.2 | 12.7 | 3.7 | 1.0 |
| LMIC | Beef | Food | Per person | 8.4 |  | 29.6 | | 5.7 | −11.9 | 30.0 | 30.4 |
| All | Sheep | Food | Per person | 2.5 |  | 43.7 | | 25.4 | 10.6 | 43.3 | 42.9 |
| HIC | Sheep | Food | Per person | 2.1 |  | 27.8 | | 42.8 | 57.7 | 21.5 | 15.7 |
| LMIC | Sheep | Food | Per person | 2.5 |  | 45.7 | | 22.6 | 3.3 | 46.1 | 46.5 |
| All | Pork | Food | Per person | 15.3 |  | −7.1 | | −14.9 | −21.3 | −8.0 | −8.8 |
| HIC | Pork | Food | Per person | 31.0 |  | 0.0 | | 0.5 | 1.0 | −4.4 | −8.1 |
| LMIC | Pork | Food | Per person | 12.4 |  | −8.2 | | −19.7 | −29.4 | −7.7 | −7.3 |
| All | Red meat total | Protein | Per person | 4.6 |  | 10.1 | | −2.0 | −11.2 | 9.4 | 8.7 |
| HIC | Red meat total | Protein | Per person | 9.0 |  | 4.3 | | 6.8 | 8.9 | 0.5 | −2.8 |
| LMIC | Red meat total | Protein | Per person | 3.8 |  | 14.5 | | −3.7 | −17.9 | 14.9 | 15.3 |
|  |  |  |  |  |  |  | |  |  |  |  |
| All | Beef | Food | Total | 83,496 |  | 44.4 | | 26.9 | 14.2 | 43.6 | 42.9 |
| HIC | Beef | Food | Total | 29,395 |  | 19.1 | | 23.0 | 25.9 | 15.7 | 12.8 |
| LMIC | Beef | Food | Total | 54,100 |  | 58.2 | | 29.0 | 7.5 | 58.7 | 59.1 |
| All | Sheep | Food | Total | 18,699 |  | 73.1 | | 51.1 | 33.3 | 72.6 | 72.1 |
| HIC | Sheep | Food | Total | 2,524 |  | 42.7 | | 59.4 | 76.1 | 35.6 | 29.2 |
| LMIC | Sheep | Food | Total | 16,175 |  | 77.9 | | 49.7 | 26.1 | 78.4 | 78.8 |
| All | Pork | Food | Total | 116,508 |  | 11.9 | | 2.5 | −5.2 | 10.8 | 9.8 |
| HIC | Pork | Food | Total | 36,664 |  | 11.6 | | 12.2 | 12.8 | 6.7 | 2.6 |
| LMIC | Pork | Food | Total | 79,844 |  | 12.1 | | −2.0 | −13.8 | 12.7 | 13.1 |
| All | Red meat total | Protein | Total | 34,871 |  | 32.6 | | 18.1 | 7.0 | 31.8 | 31.0 |
| HIC | Red meat total | Protein | Total | 10,663 |  | 16.4 | | 19.3 | 21.6 | 12.2 | 8.5 |
| LMIC | Red meat total | Protein | Total | 24,207 |  | 39.8 | | 17.5 | 0.2 | 40.3 | 40.8 |

Notes: Per person demand in kg and total demand in thousands metric tons. Projected data simulated using income and population from shared socioeconomic pathway 2. Average per person demand in 2020 reference case is total demand from all countries in a country group divided by total population from all countries in a country group. All = all countries in the world, HIC = high-income countries, LMIC = low- and middle-income countries.

**Table SI.5**

Range of percent change in demand for red meat in red meat income elasticities scenarios

| Country group | livestock-derived food | Demand type |  |  | Scenario for change in income elasticity trajectory | | | | |
| --- | --- | --- | --- | --- | --- | --- | --- | --- | --- |
|  |  |  |  |  | Reference case | 50% lower all countries | 100% lower all countries | 50% lower HIC only | 100% lower HI only |
|  |  |  | Reference case average demand in 2020 |  | Range of percent change in demand 2020 to 2050 | | | | |
| HIC | Beef | Per person | 3–44 |  | −1–95 | −5–73 | −27–51 | −8–69 | −31–46 |
| HIC | Sheep | Per person | 0–22 |  | 0–107 | 0–119 | 0–128 | −1–80 | −21–57 |
| HIC | Pork | Per person | 3–66 |  | −8–184 | −9–123 | −28–75 | −11–118 | −28–67 |
| LMIC | Beef | Per person | 0–58 |  | −8–323 | −17–120 | −54–38 | −8–325 | −7–328 |
| LMIC | Sheep | Per person | 0–60 |  | 4–200 | −5–100 | −23–97 | 4–201 | 5–202 |
| LMIC | Pork | Per person | 0–42 |  | −6–238 | −18–76 | −59–41 | −5–241 | −4–244 |
|  |  |  |  |  |  |  |  |  |  |
| HIC | Beef | Total | 2–14,880 |  | −9–114 | −9–89 | −32–78 | −12–85 | −35–60 |
| HIC | Sheep | Total | 0–359 |  | −2–126 | 2–139 | −6–150 | −7–97 | −24–72 |
| HIC | Pork | Total | 5–9,550 |  | −6–210 | −9–144 | −32–91 | −14–138 | −33–83 |
| LMIC | Beef | Total | 0–12,352 |  | −22–597 | −28–260 | −45–133 | −22–601 | −22–604 |
| LMIC | Sheep | Total | 0–6,251 |  | 0–373 | −14–233 | −35–185 | 1–374 | 1–376 |
| LMIC | Pork | Total | 0–57,604 |  | −18–475 | −28–238 | −51–99 | −17–480 | −17–484 |

Notes: Range reported as minimum to maximum. Projected data simulated using income and population from shared socioeconomic pathway 2. Column 4 absolute per person demand in kg and absolute total demand in thousand metric tons. HIC = high-income countries, LMIC = low- and middle-income countries.

**Table SI.6**

Annual per person demand for red meat protein in 2020 and percent change in demand (2020 to 2050) under changes in red meat income elasticities

| Country group |  |  | Scenario for change in income elasticity trajectory | | | | | |
| --- | --- | --- | --- | --- | --- | --- | --- | --- |
|  |  |  | Reference case (no change) | 50% lower all countries | 100% lower all countries | 50% lower HIC only | 100% lower HIC only | Elasticity equals −0.2 in 2050 in all HIC only |
|  | Reference case average per person demand in 2020 (kg) |  | Percent change in per person demand 2020 to 2050 | | | | | |
| All | 4.6 |  | 10.1 | −2.0 | −11.2 | 9.4 | 8.7 | 7.4 |
|  |  |  |  |  |  |  |  |  |
| HIC | 9.0 |  | 4.3 | 6.8 | 8.9 | 0.5 | −2.8 | −10.1 |
|  |  |  |  |  |  |  |  |  |
| LMIC | 3.8 |  | 14.5 | −3.7 | −17.9 | 14.9 | 15.3 | 16.3 |

Notes: Data in columns 3 to 8 are percent changes between 2020 and 2050 in each of the six scenarios listed in the second row. Table 3 also lists the results from the first five scenarios (columns 3 to 7), and they are listed here for comparison purposes. Demand is in protein and is summed over beef, sheep, and pork. Projected data simulated using income and population from shared socioeconomic pathway two. Average in 2020 reference case is demand from all countries in a country group divided by total population from all countries in a country group. All = all countries in the world, HIC = high-income countries, LMIC = low- and middle-income countries.

## SI figures


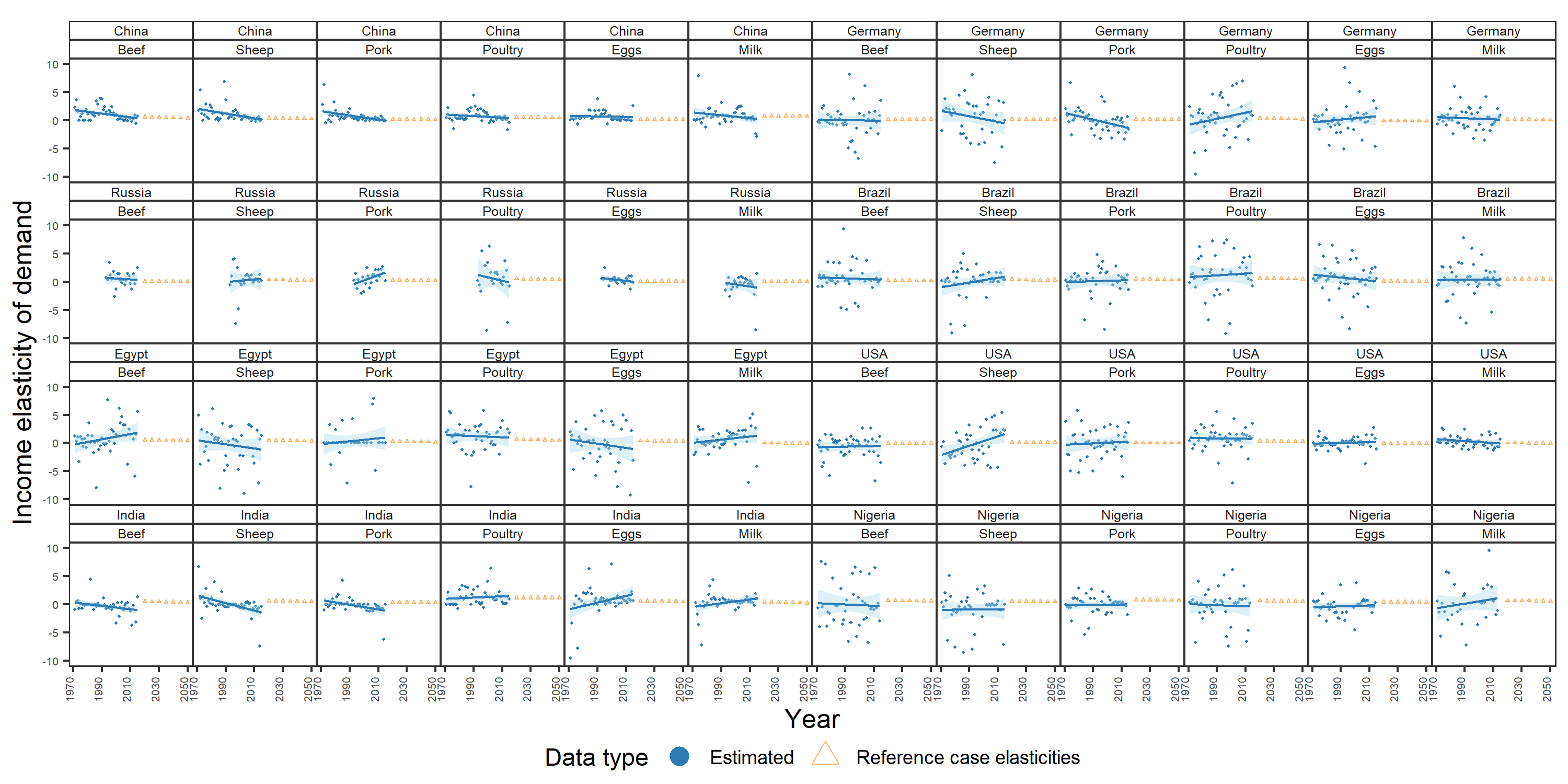


**Fig. SI.1.** Income elasticity of demand for six livestock-derived foods in eight select countries. The data labelled Estimated are our estimates of the income elasticity of demand and are based on the point elasticity of demand formula. Solid lines are linear regression lines. The data labelled Reference case elasticities are parameters in the model and are our assumed exogenous parameters. Y-axis truncated at −10 and 10 for figure clarity; however, the linear regressions use all the Estimated data points. The blue bands around each regression line are the 95% confidence intervals. Additional details on the elasticities in Fig. SI.1 are in the text directly below Fig. SI.1.

The values for the data reported as Estimated in Fig. SI.1 are for the income elasticity of demand and were estimated using the following formula: $\varepsilon_{t}=\frac{I_{t}}{D_{t}}\times\frac{D_{t}-D_{t-1}}{I_{t}-I_{t-1}}$, where $\varepsilon_{t}$is the income elasticity of demand in time period *t*, *D* is per person quantity demand, and *I* is per person income. If the time period *t* was for the calendar year 2000, then *t−*1 would be the calendar year 1999. The elasticity formula above is derived from the standard economic definition of the income elasticity of demand (De Wolff, 1941; Pindyck and Rubinfeld, 2015), which typically takes the form: $\varepsilon=\frac{I}{D}\times\frac{\Delta D}{\Delta I}$, where the symbol $\Delta$ is the Greek capital letter *delta* and often means “the change in” (Pindyck and Rubinfeld, 2015). To estimate the income elasticity of demand (the data type labelled as Estimated in Fig. SI.1) we used per person quantity demanded of each food and per person income from FAO (2020) Food Balance Sheets and Marco-Statistics. We used a yearly time step to derive the change in demand or income because FAO data are reported on a yearly time step. The following FAO item names (item code) were used for the quantity demanded: beef = Bovine Meat (2731), 962 sheep = Mutton & Goat meat (2732), pork = Pigmeat (2733), poultry = Poultry Meat (2734), eggs = Eggs (2744), 963 milk = Milk - Excluding Butter (2848).The eight countries reported in Fig. SI.1 are the countries within a region that had the largest projected total demand for protein from red meat in 2050 using income and population from SSP2 and reference case elasticities. These countries were (with percent contribution of country to region demand) 1) China in East Asia & Pacific (72%); 2) Germany in Europe (15%); 3) Russia in Former Soviet Union (48%); 4) Brazil in Latin America & Caribbean (41%); 5) Egypt in Middle East & North Africa (22%); 6) USA in North America (91%); 7) India in South Asia (47%); and 8) Nigeria in sub-Saharan Africa (15%).


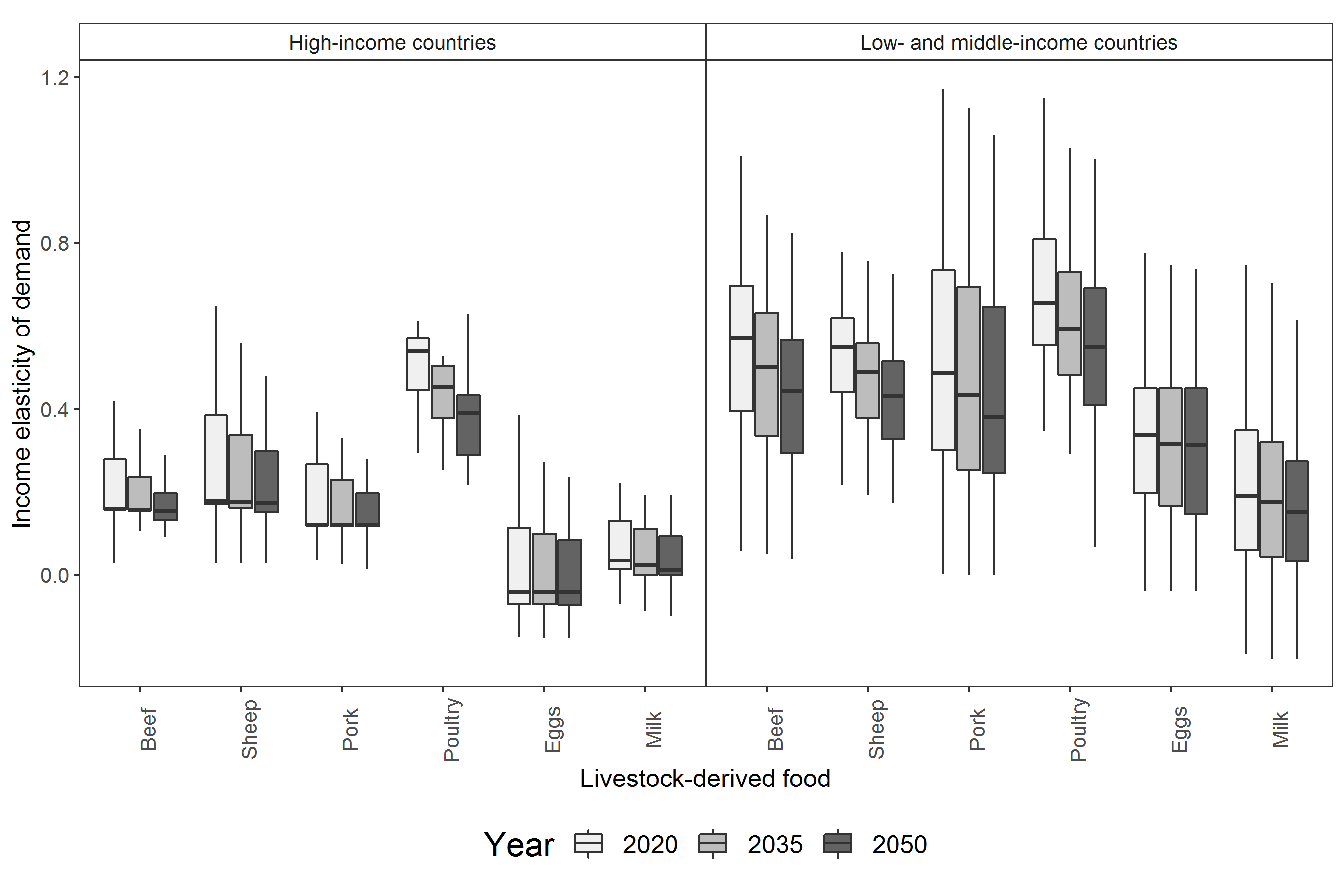
**Fig. SI.2.** Income elasticity of demand for six livestock-derived foods by income group. Countries allocated to an income group using World Bank’s Country Group classification. Boxes indicate the interquartile range (IQR). The upper whisker extends from the third quartile upper hinge of the box to the largest value no further than 1.5 × IQR from the upper hinge. The lower whisker extends from the first quartile lower hinge of the box to the smallest value at most 1.5 × IQR from the lower hinge. Outliers are not plotted for clarity. The line dividing each box shows the median.


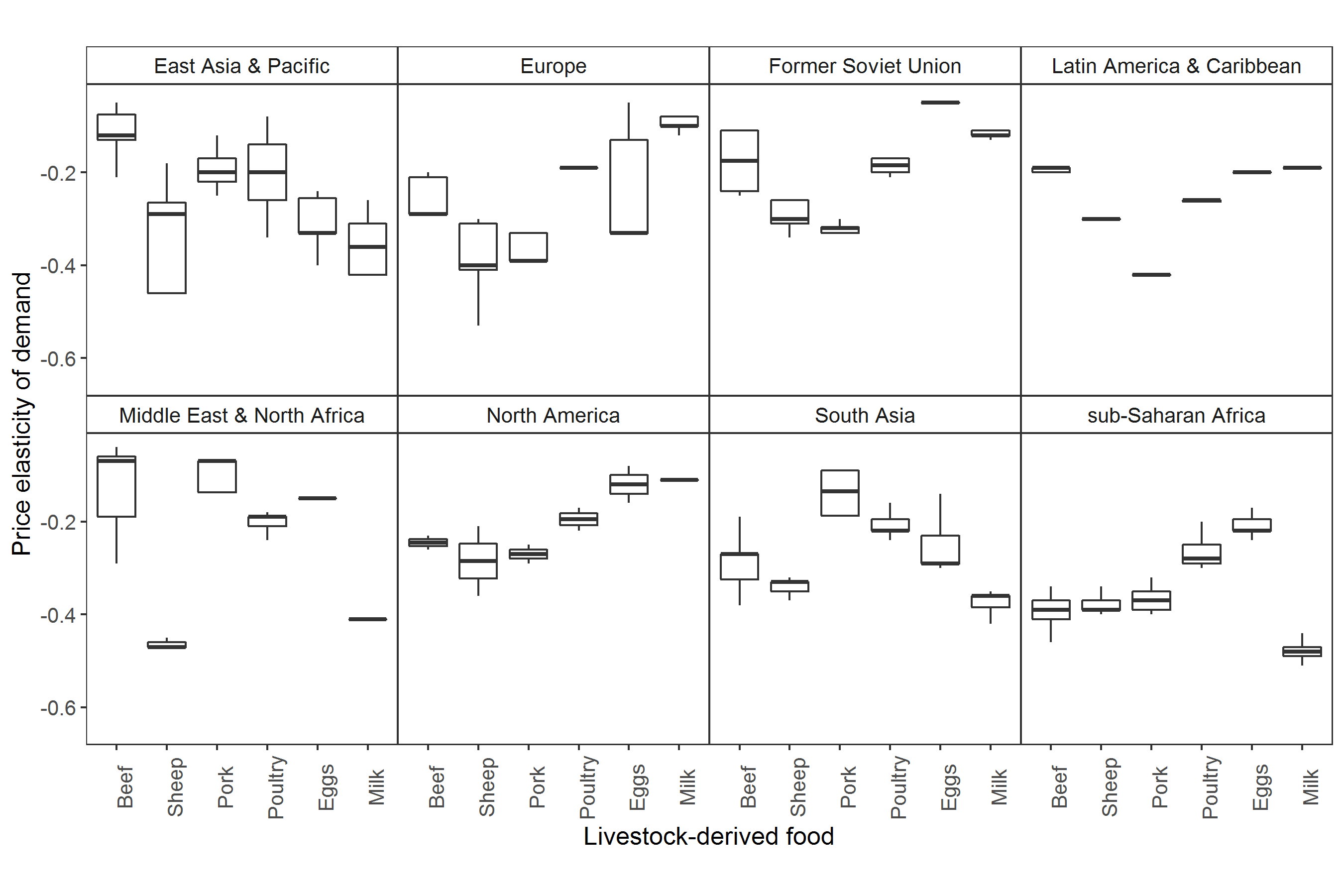


**Fig. SI.3.** Price elasticity of demand for six livestock-derived foods by region. Values are the same every year. Boxes indicate the interquartile range (IQR). The upper whisker extends from the third quartile upper hinge of the box to the largest value no further than 1.5 × IQR from the upper hinge. The lower whisker extends from the first quartile lower hinge of the box to the smallest value at most 1.5 × IQR from the lower hinge. Outliers are not plotted for clarity. The line dividing each box shows the median.


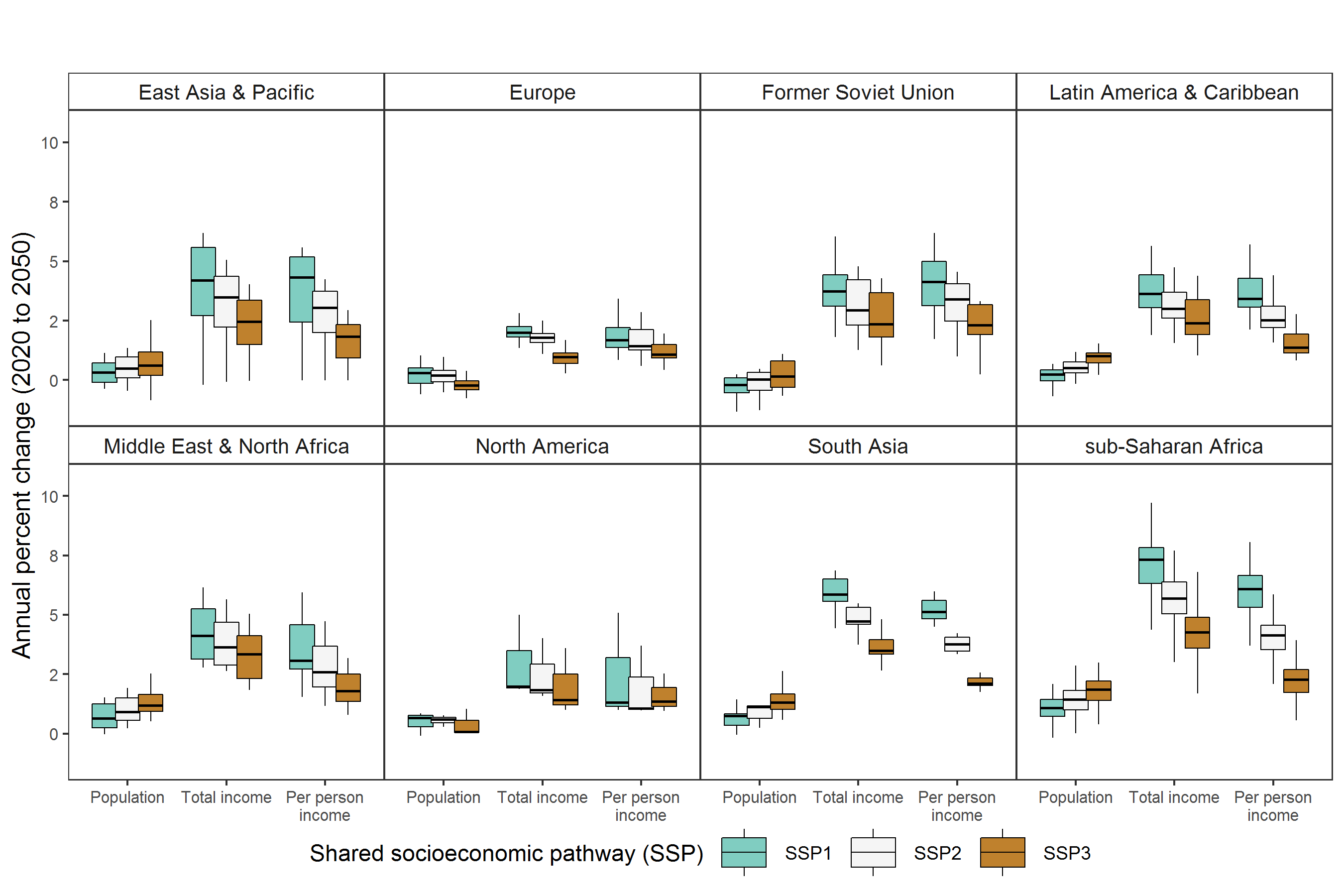


**Fig. SI.4.** Annual percent change (2020 to 2050) in country-scale human population, total income, and per person income in three shared socioeconomic pathways (SSP) by region. Boxes indicate the interquartile range (IQR). The upper whisker extends from the third quartile upper hinge of the box to the largest value no further than 1.5 × IQR from the upper hinge. The lower whisker extends from the first quartile lower hinge of the box to the smallest value at most 1.5 × IQR from the lower hinge. Outliers are not plotted for clarity. The line dividing each box shows the median.


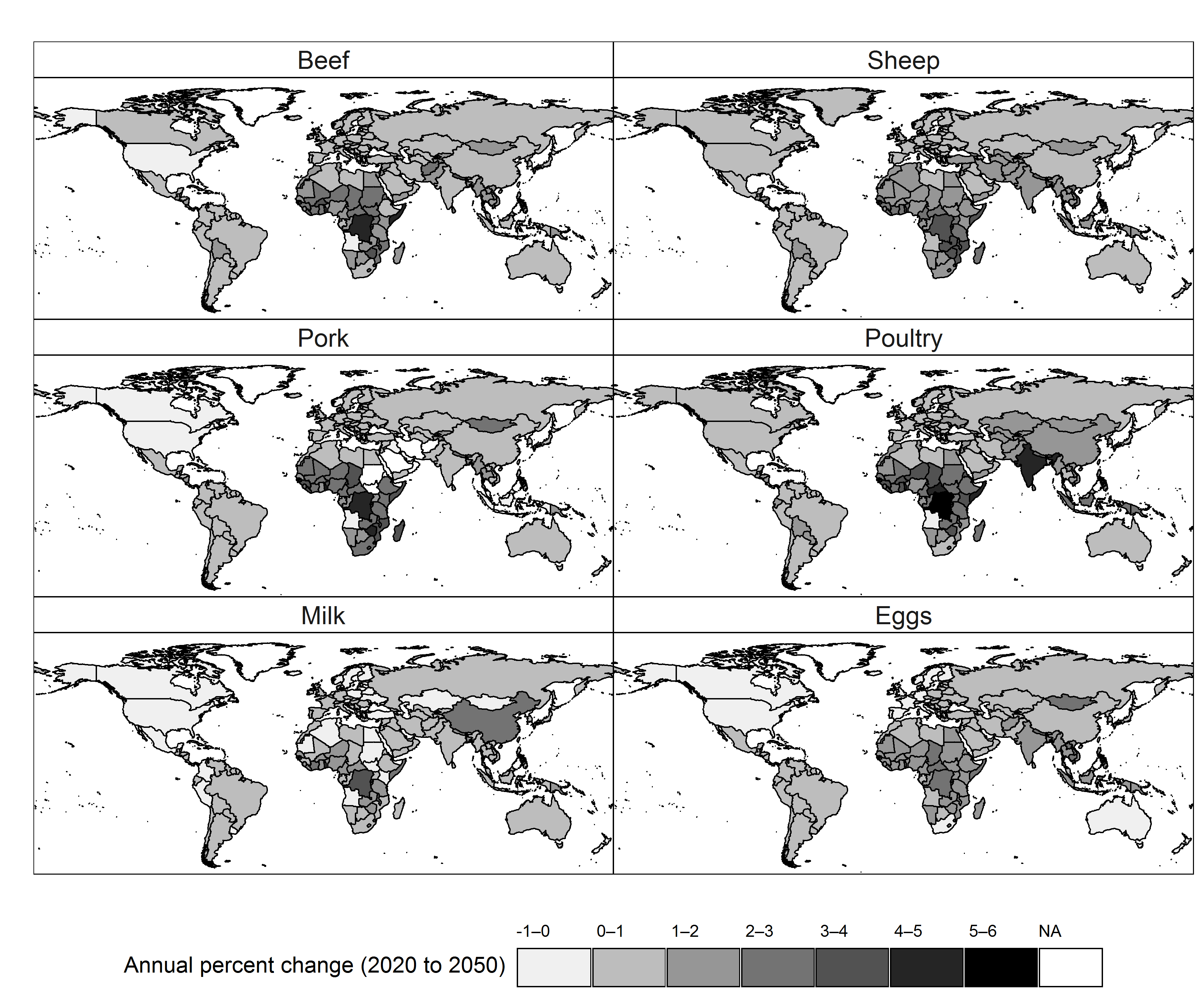


**Fig. SI.5.** Simulated country-scale annual percent change in per person demand for livestock-derived foods between 2020 and 2050 using income and population from shared socioeconomic pathway 2 and the reference case elasticities. Sheep includes sheep and goat meat. Percent change based on compound annual growth rate.


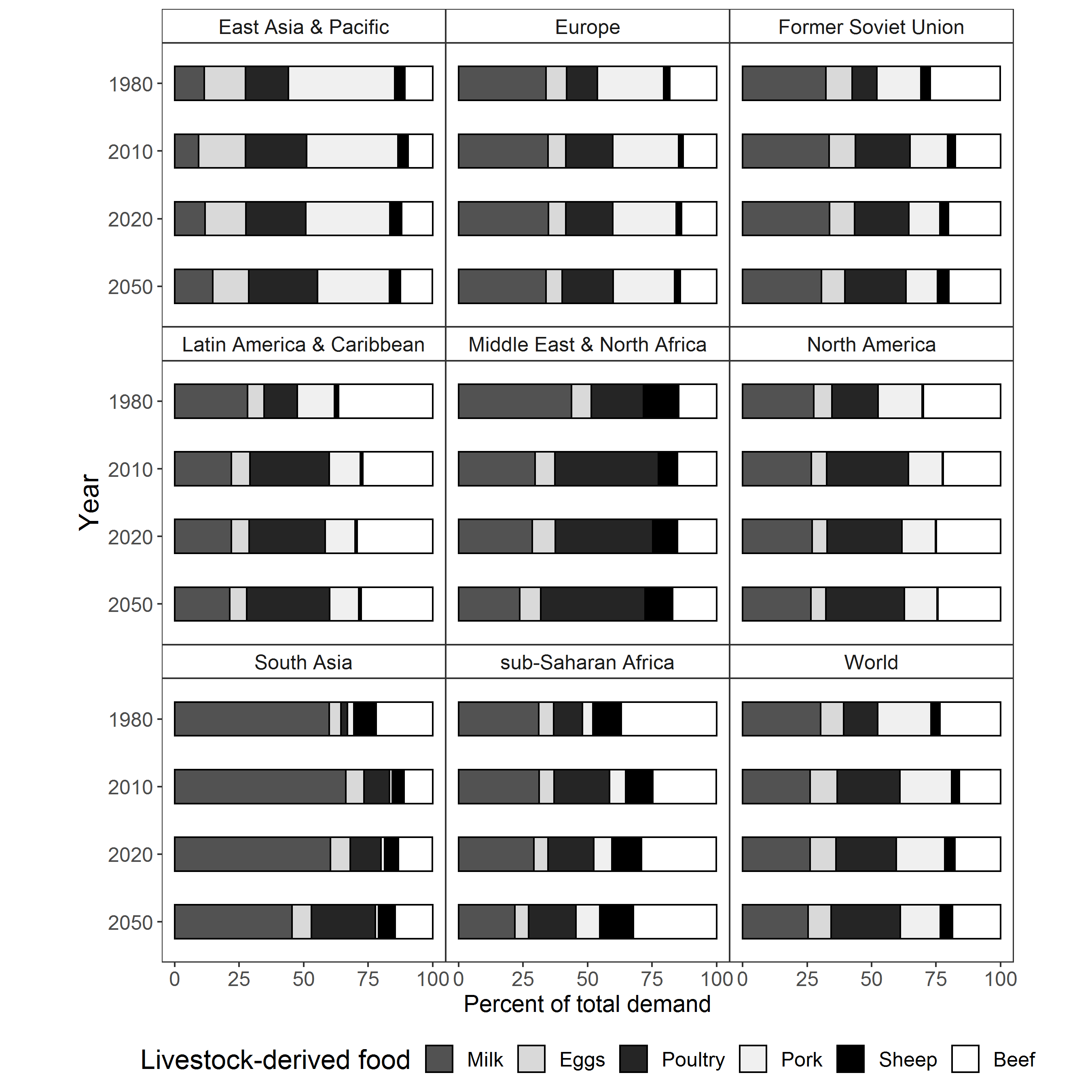


**Fig. SI.6.** Contribution of individual livestock-derived foods to per person protein demand by region and year. 1980 and 2010 are historical data from Food Balance Sheets (FAO, 2020). 2020 and 2050 are projected data simulated using income and population from shared socioeconomic pathway 2 and the reference case elasticities.


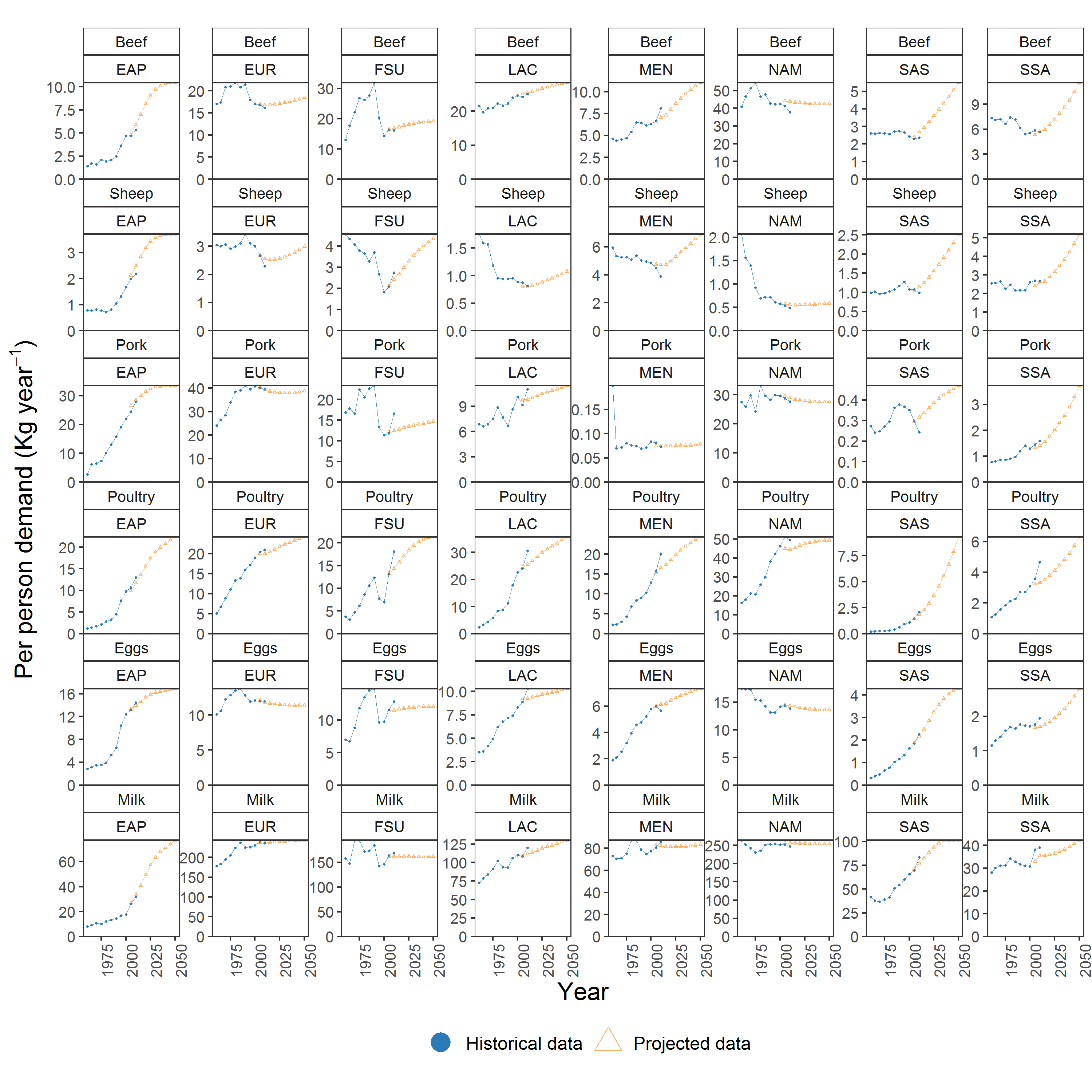


**Fig. SI.7**. Historical and projected data for per person demand for livestock-derived foods. Historical data from Food Balance Sheets (FAO, 2020). Projected data simulated using income and population from shared socioeconomic pathway 2 and the reference case elasticities. EAP = East Asia & Pacific, EUR = Europe, FSU = Former Soviet Union, LAC = Latin America & Caribbean, MEN = Middle East & North Africa, NAM = North America, SAS = South Asia, SSA = sub-Saharan Africa.


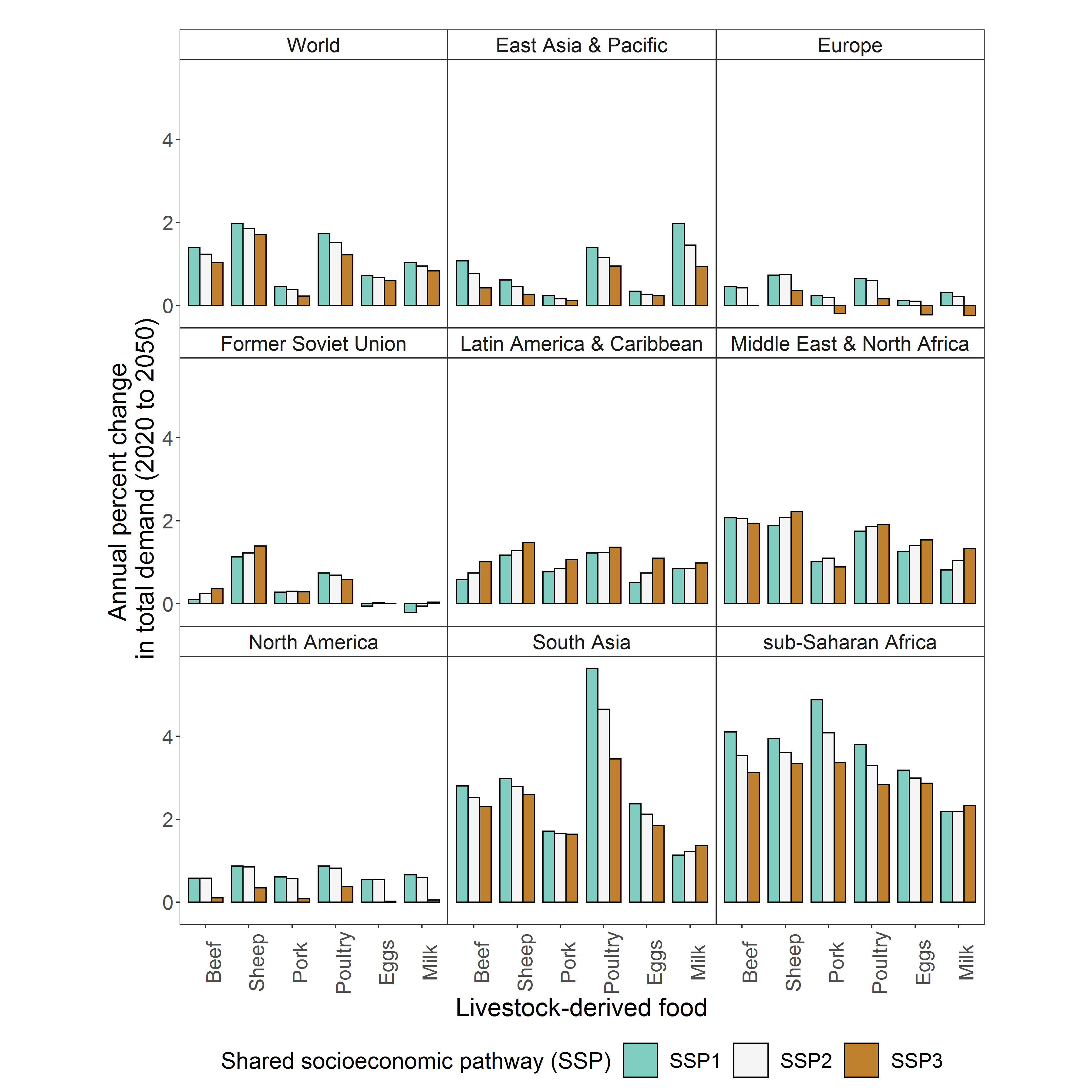


**Fig. SI.8.** Changes in total demand for livestock-derived foods by region. Projected data simulated using income and population from shared socioeconomic pathway 2 and the reference case elasticities. Percent changes are based on compound annual growth rate.


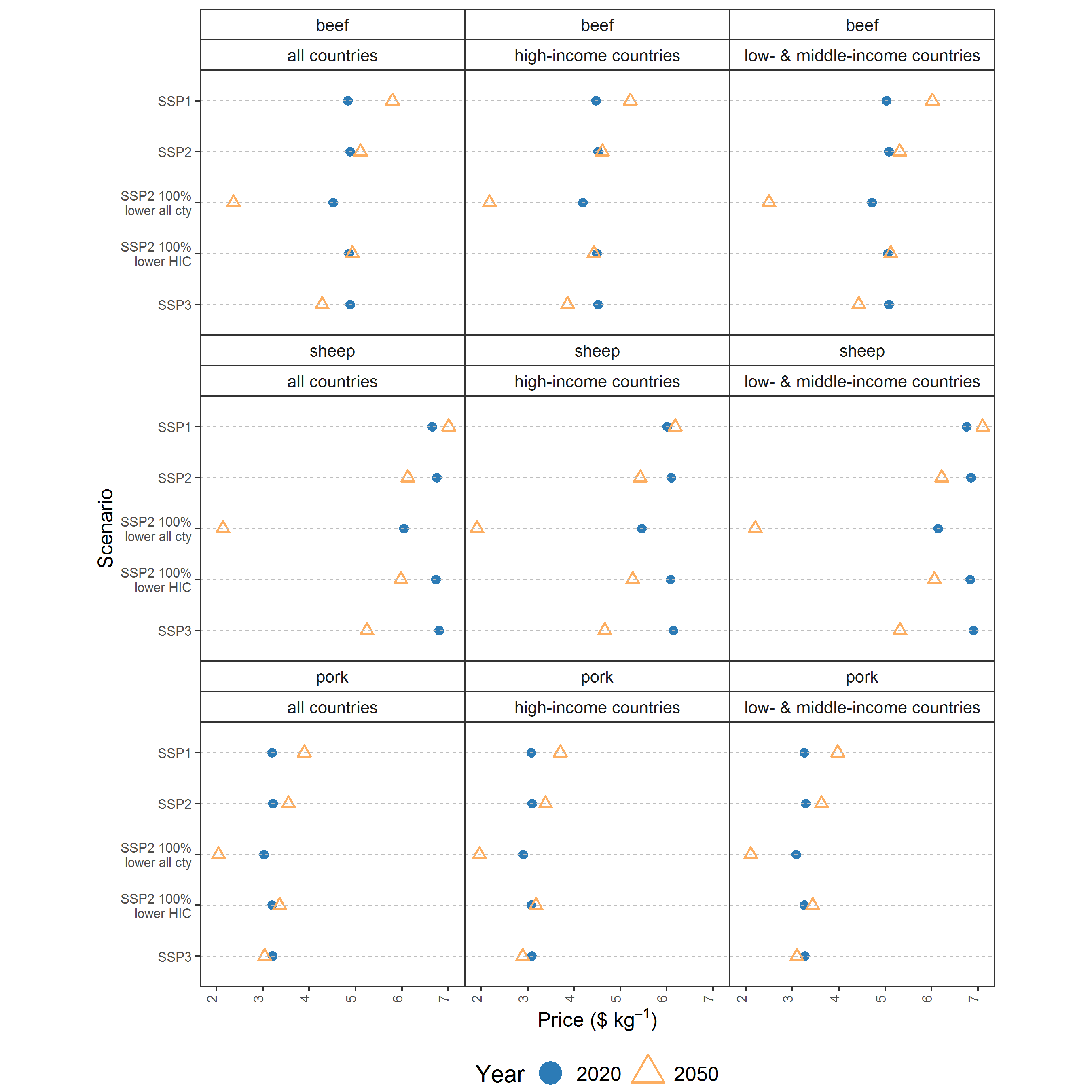


**Fig. SI.9.** Simulated red meat prices. Projected data simulated using income and population from three shared socioeconomic pathways (SSP). HIC = high-income countries and cty = countries. 100% lower means the income elasticity of demand for each of the three red meats linearly declines so that in 2050 its values are 100% less than the reference case in 2050.


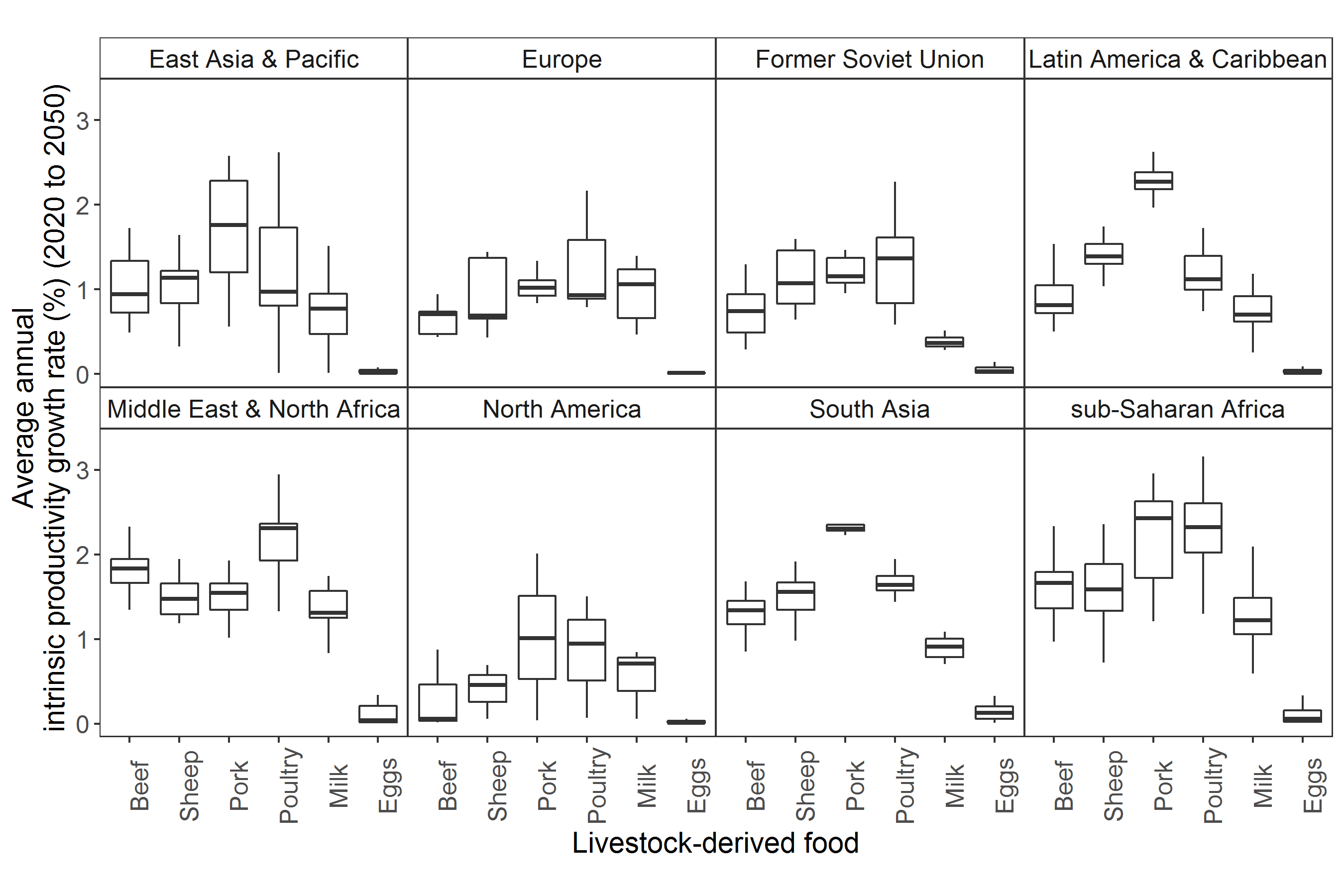


**Fig. SI.10.** Average annual (between 2020 to 2050) country-specific intrinsic productivity growth rate for six livestock-derived foods by region. Boxes indicate the interquartile range (IQR). The upper whisker extends from the third quartile upper hinge of the box to the largest value no further than 1.5 × IQR from the upper hinge. The lower whisker extends from the first quartile lower hinge of the box to the smallest value at most 1.5 × IQR from the lower hinge. Outliers are not plotted for clarity. The line dividing each box shows the median.


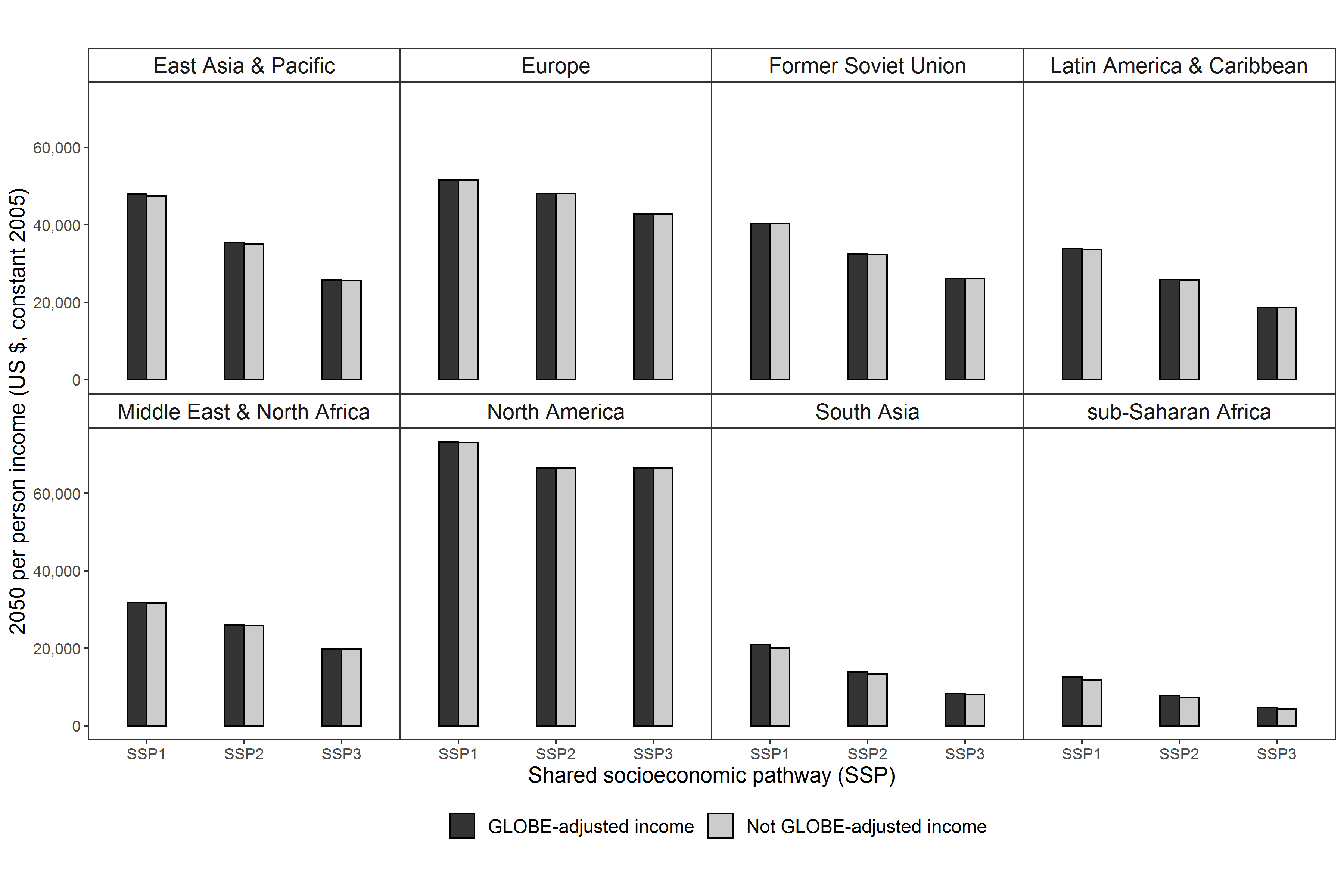


**Fig. SI.11.** Per person income in 2050 with and without GLOBE-adjusted income by region. Per person income is total income from all countries in a region divided by total population from all countries in a region.

## SI references

Aguiar, A., Chepeliev, M., Corong, E.L., McDougall, R., van der Mensbrugghe, D., 2019. The GTAP Data Base: Version 10. Journal of Global Economic Analysis 4, 1-27.

Alexandratos, N., 1999. World food and agriculture: Outlook for the medium and longer term. Proceedings of the National Academy of Sciences 96, 5908-5914.

Beach, R.H., Sulser, T.B., Crimmins, A., Cenacchi, N., Cole, J., Fukagawa, N.K., Mason-D'Croz, D., Myers, S., Sarofim, M.C., Smith, M., Ziska, L.H., 2019. Combining the effects of increased atmospheric carbon dioxide on protein, iron, and zinc availability and projected climate change on global diets: a modelling study. The Lancet Planetary Health 3, e307-e317.

Brooks, K., Place, F., 2019. Global food systems: Can foresight learn from hindsight? Global Food Security 20, 66-71.

Cooley, T.F., 1997. Calibrated models. Oxford Rev. Econ. Pol. 13, 55-69.

De Wolff, P., 1941. Income Elasticity of Demand, a Micro-Economic and a Macro-Economic Interpretation. The Economic Journal 51, 140-145.

Delzeit, R., Beach, R., Bibas, R., Britz, W., Chateau, J., Freund, F., Lefevre, J., Schuenemann, F., Sulser, T., Valin, H., Van Ruijven, B., Weitzel, M., Willenbockel, D., Wojtowicz, K., 2020. Linking Global CGE models with Sectoral Models to Generate Baseline Scenarios: Approaches, Challenges, and Opportunities. Journal of Global Economic Analysis 5, 162-195.

FAO, 2020. FAOSTAT Data. http://www.fao.org/faostat/en/#data [Accessed 28 January, 2021]

Hertel, T., McDougall, R., Narayanan, B., Aguiar, A., 2014. GTAP 8 Data Base Documentation - Chapter 14 Behavioral Parameters (Center for Global Trade Analysis). Global Trade Analysis Project (GTAP), Purdue University, West Lafayette, IN.

Jones, C., Hughes, J., Bellouin, N., Hardiman, S., Jones, G., Knight, J., Liddicoat, S., O'Connor, F., Andres, R.J., Bell, C., 2011. The HadGEM2-ES implementation of CMIP5 centennial simulations. Geoscientific Model Development 4, 543-570.

Just, R.E., 2001. Addressing the Changing Nature of Uncertainty in Agriculture. American Journal of Agricultural Economics 83, 1131-1153.

Knight, F.H., 1921. Risk, uncertainty and proﬁt. Hart, Schaffner & Marx; Houghton Mifflin Co., Boston, MA.

Mason-D'Croz, D., Sulser, T.B., Wiebe, K., Rosegrant, M.W., Lowder, S.K., Nin-Pratt, A., Willenbockel, D., Robinson, S., Zhu, T., Cenacchi, N., Dunston, S., Robertson, R.D., 2019. Agricultural investments and hunger in Africa modeling potential contributions to SDG2 – Zero Hunger. World Development 116, 38-53.

Moss, R.H., Edmonds, J.A., Hibbard, K.A., Manning, M.R., Rose, S.K., van Vuuren, D.P., Carter, T.R., Emori, S., Kainuma, M., Kram, T., Meehl, G.A., Mitchell, J.F.B., Nakicenovic, N., Riahi, K., Smith, S.J., Stouffer, R.J., Thomson, A.M., Weyant, J.P., Wilbanks, T.J., 2010. The next generation of scenarios for climate change research and assessment. Nature 463, 747-756.

O’Neill, B.C., Kriegler, E., Riahi, K., Ebi, K.L., Hallegatte, S., Carter, T.R., Mathur, R., van Vuuren, D.P., 2014. A new scenario framework for climate change research: the concept of shared socioeconomic pathways. Climatic Change 122, 387-400.

Pindyck, R., Rubinfeld, D., 2015. Microeconomics, Global Edition. Pearson, Boston.

Reilly, M., Willenbockel, D., 2010. Managing uncertainty: a review of food system scenario analysis and modelling. Philosophical Transactions of the Royal Society B: Biological Sciences 365, 3049-3063.

Ringler, C., Willenbockel, D., Perez, N., Rosegrant, M., Zhu, T., Matthews, N., 2016. Global linkages among energy, food and water: an economic assessment. Journal of Environmental Studies and Sciences 6, 161-171.

Robinson, S., Mason D'Croz, D., Islam, S., Sulser, T.B., Robertson, R.D., Zhu, T., Gueneau, A., Pitois, G., Rosegrant, M.W., 2015. The International Model for Policy Analysis of Agricultural Commodities and Trade (IMPACT): Model description for version 3. International Food Policy Research Institute (IFPRI), Washington, D.C. http://ebrary.ifpri.org/cdm/ref/collection/p15738coll2/id/129825 [Accessed 28 January, 2021]

Smith, M.R., Micha, R., Golden, C.D., Mozaffarian, D., Myers, S.S., 2016. Global Expanded Nutrient Supply (GENuS) Model: A New Method for Estimating the Global Dietary Supply of Nutrients. Plos One 11, e0146976.

Thornton, P.K., 2010. Livestock production: recent trends, future prospects. Philosophical Transactions of the Royal Society B: Biological Sciences 365, 2853-2867.

USDA, 1998. Commodity and food elasticities. https://www.ers.usda.gov/data-products/commodity-and-food-elasticities/ [Accessed 28 January, 2021]

Uthes, S., Fricke, K., König, H., Zander, P., van Ittersum, M., Sieber, S., Helming, K., Piorr, A., Müller, K., 2010. Policy relevance of three integrated assessment tools—A comparison with specific reference to agricultural policies. Ecological Modelling 221, 2136-2152.

Valin, H., Sands, R.D., van der Mensbrugghe, D., Nelson, G.C., Ahammad, H., Blanc, E., Bodirsky, B., Fujimori, S., Hasegawa, T., Havlik, P., Heyhoe, E., Kyle, P., Mason-D'Croz, D., Paltsev, S., Rolinski, S., Tabeau, A., van Meijl, H., von Lampe, M., Willenbockel, D., 2014. The future of food demand: understanding differences in global economic models. Agricultural Economics 45, 51-67.

Varian, H.R., 1992. Microeconomic Analysis. Third Edition. W.W Norton & Company, New York.

Verburg, P.H., Rounsevell, M.D.A., Veldkamp, A., 2006. Scenario-based studies of future land use in Europe. Agriculture, Ecosystems & Environment 114, 1-6.

von Lampe, M., Willenbockel, D., Ahammad, H., Blanc, E., Cai, Y., Calvin, K., Fujimori, S., Hasegawa, T., Havlik, P., Heyhoe, E., Kyle, P., Lotze-Campen, H., Mason d'Croz, D., Nelson, G.C., Sands, R.D., Schmitz, C., Tabeau, A., Valin, H., van der Mensbrugghe, D., van Meijl, H., 2014. Why do global long-term scenarios for agriculture differ? An overview of the AgMIP Global Economic Model Intercomparison. Agricultural Economics 45, 3-20.

Wiebe, K., Lotze-Campen, H., Sands, R., Tabeau, A., van der Mensbrugghe, D., Biewald, A., Bodirsky, B., Islam, S., Kavallari, A., Mason-D’Croz, D., Müller, C., Popp, A., Robertson, R., Robinson, S., van Meijl, H., Willenbockel, D., 2015. Climate change impacts on agriculture in 2050 under a range of plausible socioeconomic and emissions scenarios. Environmental Research Letters 10, 085010.

Willenbockel, D., Robinson, S., Mason-D’Croz, D., Rosegrant, M., Sulser, T., Dunston, S., Cenacchi, N., 2018. Dynamic Computable General Equilibrium Simulations in Support of Quantitative Foresight Modeling to Inform the CGIAR Research Portfolio. International Food Policy Research Institute (IFPRI), Washington, DC. http://ebrary.ifpri.org/cdm/ref/collection/p15738coll2/id/132757 [Accessed 28 January, 2021]
